# VDR Signaling via the Enzyme NAT2 Inhibits Colorectal Cancer Progression

**DOI:** 10.3389/fphar.2021.727704

**Published:** 2021-11-16

**Authors:** Chaojun Zhu, Zihuan Wang, Jianqun Cai, Chunqiu Pan, Simin Lin, Yue Zhang, Yuting Chen, Mengxin Leng, Chengcheng He, Peirong Zhou, Changjie Wu, Yuxin Fang, Qingyuan Li, Aimin Li, Side Liu, Qiuhua Lai

**Affiliations:** ^1^ Guangdong Provincial Key Laboratory of Gastroenterology, Department of Gastroenterology, Nanfang Hospital, Southern Medical University, Guangzhou, China; ^2^ Department of Emergency Medicine, Nanfang Hospital, Southern Medical University, Guangzhou, China; ^3^ Department of Gastroenterology, Third Affiliated Hospital of Guangzhou Medical University, Guangzhou, China

**Keywords:** colorectal cancer, vitamin D, VDR (vitamin D receptor), network pharmacology, NAT2 (N-acetyl transferase 2)

## Abstract

Recent epidemiological and preclinical evidence indicates that vitamin D_3_ inhibits colorectal cancer (CRC) progression, but the mechanism has not been completely elucidated. This study was designed to determine the protective effects of vitamin D_3_ and identify crucial targets and regulatory mechanisms in CRC. First, we confirmed that 1,25(OH)_2_D_3_, the active form of vitamin D_3_, suppressed the aggressive phenotype of CRC *in vitro* and *in vivo*. Based on a network pharmacological analysis, N-acetyltransferase 2 (NAT2) was identified as a potential target of vitamin D_3_ against CRC. Clinical data of CRC patients from our hospital and bioinformatics analysis by online databases indicated that NAT2 was downregulated in CRC specimens and that the lower expression of NAT2 was correlated with a higher metastasis risk and lower survival rate of CRC patients. Furthermore, we found that NAT2 suppressed the proliferation and migration capacity of CRC cells, and the JAK1/STAT3 signaling pathway might be the underlying mechanism. Moreover, Western blot and immunofluorescence staining assays demonstrated that 1,25(OH)_2_D_3_ promoted NAT2 expression, and the chromatin immunoprecipitation assay indicated that the vitamin D receptor (VDR) transcriptionally regulated NAT2. These findings expand the potential uses of vitamin D_3_ against CRC and introduce VDR signaling via the enzyme NAT2 as a potential diagnostic and therapeutic target for CRC.

## Introduction

Colorectal cancer (CRC) is the third most commonly diagnosed cancer and the second most common cause of cancer death worldwide (Laversanne et al., 2020; Sung et al., 2021). Although the 5-year survival rate of CRC has been increasing in recent years (Brenner et al., 2014; Laversanne et al., 2020), poor prognosis always brings a heavy burden to patients and health care. Both genetic and environmental factors are known to be involved in CRC initiation and progression (Lichtenstein et al., 2000; Brenner et al., 2014).

Since Garland et al. proposed that vitamin D deficiency is a risk factor for CRC (Garland and Garland, 1980), a number of epidemiological studies, clinical trials, and mechanism research studies have been initiated and revealed that vitamin D exerts antitumor actions (Lamprecht and Lipkin, 2003). CRC is the cancer that is most frequently related to vitamin D deficiency in aspects of incidence and prognosis (Ng et al., 2011; Ferrer-Mayorga et al., 2019; Wu et al., 2019). Vitamin D intake and blood 25(OH)D levels are inversely associated with the risk and survival of CRC (Garland et al., 1985; Ng et al., 2008; Jenab et al., 2010; Ng et al., 2011; Zgaga et al., 2014; McCullough et al., 2019). The predominant active metabolite of vitamin D is 1α,25-dihydroxyvitamin D_3_ (1,25(OH)_2_D_3_) (also known as calcitriol), which inhibits proliferation and angiogenesis in CRC (Feldman et al., 2014; Ji et al., 2020). 1,25(OH)_2_D_3_ actions are mainly mediated by the vitamin D receptor (VDR), a member of the superfamily of nuclear receptors that plays the role of transcriptional regulation by binding to vitamin D response elements (VDREs) on target genes (Feldman et al., 2014; Wu et al., 2019). Studies have shown that the VDR expression level is related to the degree of differentiation of CRC cells, and Pei-Shan Hu et al. demonstrated that the acidic tumor microenvironment involves in VDR-SOX2 signaling and promotes CRC stemness (Hu et al., 2020). Besides, 1,25(OH)_2_D_3_ inhibited the protumoral activation of patient-derived colon normal fibroblasts (NFs) and cancer-associated fibroblasts (CAFs) and a 1,25(OH)_2_D_3_-associated gene signature imposed in CAFs correlated with a better prognosis in CRC (Ferrer-Mayorga et al., 2017). Nevertheless, there is still a lack of sufficient and potent evidence from prospective clinical trials at present, and nonresponse to vitamin D may result from molecular mutation and regulation of the cancer-associated pathway (Barry et al., 2017; Bhasin et al., 2018). Thus, it is essential to further reveal the biological mechanisms illustrating the antitumor role of vitamin D_3_ in CRC.

Network pharmacological analysis, a useful and integrative approach emerging recently *in silico*, is widely used to identify candidate targets for drug effects against diseases and systematically explore biological functions and molecular mechanisms (Hopkins, 2008; Ma'ayan et al., 2014; Wang et al., 2020). With network pharmacological analysis, we identified that N-acetyltransferase 2 (NAT2), which is an enzyme that activates or deactivates arylamine and hydrazine of drugs and carcinogens (Hickman et al., 1998), is one of 29 intersecting targets of vitamin D_3_ against CRC. However, NAT2 expression profiling and molecular function in CRC have not been clarified.

In this study, we aimed to verify the hypothesis that 1,25(OH)_2_D_3_ regulates VDR signaling via the enzyme NAT2 and restrains the progression of CRC by bioinformatics analysis and molecular biology experiments. Additionally, we aimed to elucidate the expression profiling of NAT2 and the effects of NAT2 on the malignant biological behavior of CRC cell lines and the potential mechanism.

## Materials and Methods

### Clinical Samples

Human colorectal cancer and adjacent normal tissues for real-time quantitative PCR (RT-qPCR) analysis and immunohistochemical (IHC) staining were obtained from patients who underwent surgery in the Department of General Surgery of Nanfang Hospital, Southern Medical University. All patients were pathologically diagnosed with colorectal cancer and had not received radiotherapy or chemotherapy before surgery. The protocol in this study was approved by the Institutional Review Board for Human Use at Nanfang Hospital. Written informed consent for this study was obtained from all patients prior to surgery.

### Cell Lines and Cell Culture

Human embryonic kidney cell line (293T), human normal colorectal cell line (FHC), and human colorectal cancer cell lines (SW620, LoVo, SW480, HT29, RKO, and HCT116) were commercially obtained from the Cell Bank of Type Culture Collection (Chinese Academy of Sciences, Shanghai, China). All cells were cultured in DMEM (Gibco, Carlsbad, United States) supplemented with 10% FBS (Gibco, Carlsbad, United States) at 37°C in a humidified atmosphere with 5% CO2. For some experiments, cells were treated with 100 nM calcitriol (MedChemExpress, New Jersey, United States) for the indicated time (the medium was changed every two or 3 days in long-time experiments).

### Cell Proliferation Assay and Colony Formation Assay

Cells treated with calcitriol or transfected with plasmid or siRNA were seeded into 96-well plates at a density of 1 × 10^3^ cells per well. For the cell proliferation assay, the cells were subsequently incubated with 10 μl of resazurin (Meilunbio, Dalian, China) per well for 2 h, and the optical density (OD) value was detected at a wavelength of 590 nm using SpectraMax M4 Multimode microplate readers (Molecular Devices, San Jose, United States). The cells were further incubated for 5 days, followed by the cell proliferation assay every day. For the colony formation assay, cells were seeded in 6-well plates at a density of 500 cells per well and incubated for approximately 10 days. The medium was replaced every 3 days. Finally, the cells were fixed with 4% paraformaldehyde and stained with crystal violet solution (Solarbio, Beijing, China) for count and analysis.

### Cell Migration Assay

Approximately 2 × 10^5^ SW480 cells or 1 × 10^5^ LoVo cells were suspended in 200 μl serum-free DMEM and placed into an 8.0-µm pore polycarbonate membrane insert (Corning, New York, United States), and the lower chamber was filled with 600 μl DMEM containing 10% FBS. After incubation for 24 h for SW480 cells or 48 h for LoVo cells, the cells were fixed with 4% paraformaldehyde and stained with crystal violet solution. Finally, we used swabs to remove the cells from the upper surface of the membranes and counted the cells on the lower surface in five high-power fields under a light microscope.

### Cell-Derived Xenograft and Drug Treatment

As previously mentioned (Lai et al., 2020), female athymic 4-week-old BALB/c nude mice were obtained from the Central Laboratory of Animal Science at Southern Medical University and maintained in a specific pathogen-free facility. For the cell-derived xenograft model, 5 × 10^6^ cells were subcutaneously injected into the left back of nude mice. One week later, the mice were randomly grouped (five mice per group), and treatment was initiated. The mice received an intraperitoneal injection of vehicle or calcitriol (50 μg/kg, three times a week) for 2 weeks. During drug treatment, tumor volume was calculated three times a week using the following formula: (L × W^2^)/2, where L and W refer to the tumor diameter along the longitudinal and transverse axes, respectively. Finally, mice were sacrificed, and subcutaneous tumors were dissected and collected for IHC staining. The animal study was reviewed and approved by the Nanfang Hospital Animal Ethics Committee (application number: NFYY-2019-87; approval date: August 11, 2019).

### Network Pharmacological Analysis and Other Bioinformatic Analysis

The expression profile of 612 colorectal samples (normal samples, 44 cases; tumor samples, 568 cases) and the corresponding clinical data were downloaded from The Cancer Genome Atlas (TCGA) database (https://portal.gdc.cancer.gov/). The expression profile of GSE17538 was downloaded from the Gene Expression Omnibus (GEO) database (https://www.ncbi.nlm.nih.gov/geo/query/acc.cgi?acc=GSE17538). R software (version: x64 3.6.1) and the edgeR package were used to screen differentially expressed genes (DEGs) in the TCGA dataset, with the standard of fold change >2 and false discovery rate (FDR) < 0.05. A volcano map of DEGs was constructed using ggplot2 and other packages.

The CRC potential target genes were predicted by six online databases: Kyoto Encyclopedia of Genes and Genomes (KEGG, https://www.kegg.jp/), Online Mendelian Inheritance in Man (OMIM, https://www.omim.org/), Therapeutic Target Database (TTD, http://db.idrblab.net/ttd/), DrugBank (https://go.drugbank.com/), GeneCards (http://www.genecards.org/), and DisGeNET (http://www.disgenet.org/home/). The detailed information of vitamin D_3_ was obtained from PubChem (https://pubchem.ncbi.nlm.nih.gov/), and the Smile number (CC(C)CCCC(C)C1CCC2C1(CCCC2=CC=C3CC(CCC3=C)O)C) and compound CID (5280795) were acquired for predicting targets of vitamin D_3_. Subsequently, vitamin D_3_–related target genes were explored from four online databases: Drug Repositioning and Adverse Reaction via Chemical-Protein Interactome (DRAR-CPI; http://cpi.bio-x.cn/), SwissTargetPrediction (http://www.swisstargetprediction.ch/), PubChem, and PharmMapper (http://www.lilab-ecust.cn/pharmmapper/). The Database for Annotation, Visualization and Integrated Discovery (DAVID) was employed to obtain the functional enrichment of the core targets. A *p*-value cutoff = 0.05 was set for enrichment, and the top 20 pathways sorted by *p*-value were used for plotting the bubble chart with R software and the ggplot2 package.

The Search Tool for the Retrieval of Interacting Genes (STRING; http://string-db.org) was utilized to predict the protein–protein intersection (PPI) network of common targets between vitamin D_3_ and CRC, and targets with a combined score >0.7 were reserved for establishing PPI network. The network was visualized by Cytoscape software (https://cytoscape.org/, version 3.7.2). In addition, ClueGO in Cytoscape software was applied for the Gene Ontology (GO) and KEGG functional enrichment analysis.

Gene Expression Profiling Interactive Analysis (GEPIA, http://gepia.cancer-pku.cn/), PROGgeneV2 (http://genomics.jefferson.edu/proggene/), and R language loaded with package survival were applied for the survival analysis. The Kaplan–Meier method was used to plot the survival curve.

R software and the limma and beeswarm packages were applied for the analysis and visualization of gene expression in tumor and normal samples and the clinicopathologic characteristics with gene expression. The Wilcoxon rank-sum or Kruskal–Wallis rank sum test was used as the significance test for comparison. The multivariate Cox regression analysis and forest plots were carried out using R software and the survival and survminer packages. Gene set enrichment analysis (GSEA) was performed using GSEA software (http://www.gsea-msigdb.org/gsea/index.jsp, version 4.1.0).

### RNA Isolation and PCR

Total RNA was extracted from tissues or cells with RNAiso Plus (TaKaRa, Japan). Reverse transcription PCR and RT-qPCR were performed using PrimeScript™ RT Master Mix (TaKaRa, Japan) and TB Green^®^ Premix Ex Taq™ (TaKaRa, Japan) following the manufacturer’s instructions. Gene expression of interest was normalized to GAPDH, and data were analyzed with the 2^–ΔΔCt^ method. The primers used are listed in the Supplementary Table S1.

### Plasmid and siRNA Construction and Transfection

To construct the NAT2 plasmid, full-length NAT2 (NM_000015) was inserted into a vector (pLV-CMV-MCS-3FLAG-IRES-Puro) (Umine, Guangzhou, China) and then transfected into SW480 or LoVo cells by Lipofectamine™ 3000 Transfection Reagent (Invitrogen, Carlsbad, United States) according to the manufacturer’s instructions. The interference of endogenous NAT2 was achieved by siRNA (GenePharma, Shanghai, China). The NAT2-targeting siRNA and negative control sequences are listed in the Supplementary Table S1. The VDR-overexpressing plasmid was constructed based on pEnter (Vigene, Jinan, China), and a full-length VDR (NM_000376) was inserted.

### Western Blot

Western blot was performed following the standard procedure. In brief, protein lysates were separated by 10% sodium dodecyl sulfate–polyacrylamide gel electrophoresis and transferred onto polyvinylidene difluoride membranes, which were then incubated with primary antibodies at 4°C overnight and with HRP-conjugated secondary antibodies (Beyotime, Shanghai, China) for 1 h at room temperature. The following primary antibodies were used: NAT2 (1:1,000, BS72649, Bioworld, Nanjing, China), VDR (1:1,000, #12550, Cell Signaling Technology, Danvers, United States), GAPDH (1; 20000, 60004-1-Ig, Proteintech, Rosemont, United States), JAK1 (1:500, A18323 ABclonal Technology, Wuhan, China), phospho-JAK1(Y1022/1023) (1:1,000, AP0530, ABclonal Technology), STAT3 (1:1,000, #4904, Cell Signaling Technology), phospho-STAT3 (1:1,000, #9145, Cell Signaling Technology), and α-tubulin (1:10,000, RM 2007, Rayantibody, Beijing, China). Finally, protein bands were incubated with a super ECL detection reagent (Yeasen, Shanghai, China) and visualized by an automatic chemiluminescence image analysis system (Tanon, Shanghai, China).

### Immunohistochemical Staining

Serial sections (3 μm in thickness) of paraffin-embedded CRC tissues were subjected to hematoxylin–eosin and IHC staining. The sections were incubated with NAT2 antibody (1:500, Bioworld), VDR antibody (1:200, Cell Signaling Technology), or Ki67 antibody (1:8,000, 27309-1-AP, Proteintech). Then antirabbit secondary antibodies (PV-6001, ZSGB-BIO, Beijing, China) and DAB chromogenic agent (ZLI-9017, ZSGB-BIO) were used according to the manufacturer’s instructions.

### Immunofluorescence Staining

As previously mentioned (Luo et al., 2020), cells were fixed with 4% paraformaldehyde for 15 min, permeabilized with 0.5% Triton X-100 for 20 min, and blocked with 2% BSA blocking solution for 30 min at room temperature. Before each step mentioned earlier, the cells were washed gently three times for 3 min. Then, the cells were incubated with primary antibodies against VDR (1:100, Cell Signaling Technology) or NAT2 (1:100, Bioworld) at 4°C overnight. After washing, the cells were incubated with IgG-Alexa Fluor 647 antibody (1:200, HA1106, HUABIO, Hangzhou, China) for 60 min at room temperature in the dark. Afterward, samples were counterstained with DAPI (P0131, Beyotime) and imaged by an Olympus fluorescence microscope.

### Chromatin Immunoprecipitation

A SimpleChIP Enzymatic Chromatin IP Kit (Magnetic Beads) (#9003, Cell Signaling Technology) was used according to the manufacturer’s instructions. For each immunoprecipitation, a total of 4 × 10^6^ SW480 cells and 1.22 μg of anti-VDR antibody (#12550, Cell Signaling Technology) were used, and the same amount of normal rabbit IgG was used as the negative control and 10 μl histone H3 rabbit antibody was used as the positive control. RT-qPCR was used to verify the binding sites between VDR and the promoter of NAT2. The data were calculated as relative enrichment against the negative control. The primers used are listed in Supplementary Table S1.

### Statistical Analysis

All statistical analyses were performed using GraphPad Prism software (version 8.0, San Diego, United States), IBM SPSS Statistics software (version 20.0, Chicago, United States), and R software (version: x64 3.6.1). Data are shown as mean ± standard deviation (SD). Student’s *t*-test and chi-square test were used to detect significance between two groups. Spearman’s correlation analysis was performed to detect the expression correlation of different genes. The log-rank test was applied for Kaplan–Meier survival analysis. A *p* value < 0.05 was considered statistically significant (in all figures: *, *p* < 0.05; **, *p* < 0.01; ***, *p* < 0.001; n.s., non-significant).

## Results

### 1,25(OH)_2_D_3_ Suppresses Malignancy of CRC *in vitro* and *in vivo*

**FIGURE 1:**
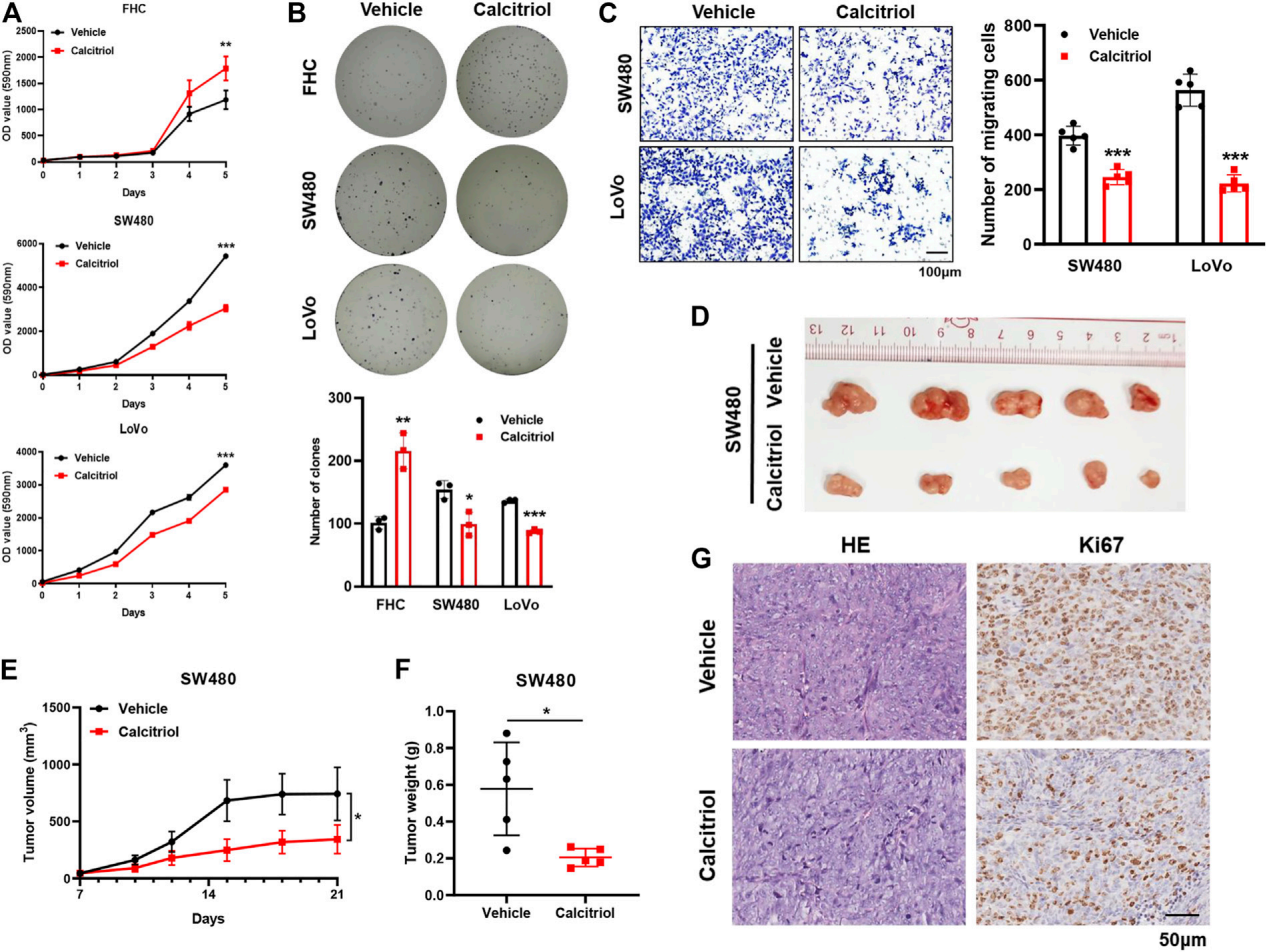
1,25(OH)_2_D_3_ suppresses the proliferation and migration capacity of CRC cells. **(A**,**B)** Cell proliferation assay **(A)** and colony formation assay **(B)** showed that calcitriol increased the proliferation capacity of FHC cells but decreased that of SW480 and LoVo cells. **(C)** Cell migration assays in SW480 and LoVo cells treated with 100 nM calcitriol or vehicle. Scale bar: 100 μm. **(D)** Tumor images of mice (*n* = 5) subcutaneously injected with SW480 cells and subsequently treated with calcitriol or vehicle. **(E)** Tumor size was measured three times a week during drug treatment. **(F)** Tumor weight was measured after 21 days. **(G)** Representative images of hematoxylin-eosin and IHC staining for Ki67 in subcutaneous tumors. Scale bar: 50 μm. Three independent experiments were performed for **(A**–**C)**. The data are presented the mean ± SD (Student’s *t* test. *, *p* < 0.05; **, *p* < 0.01; ***, *p* < 0.001).

The effects of calcitriol in CRC were determined in the colorectal cancer cell lines SW480 and LoVo and the normal colorectal cell line FHC. According to the results of the cell proliferation assay, FHC cells displayed faster growth, and SW480 and LoVo cells were inhibited slightly after treatment with 100 nM calcitriol (Figure 1A). Consistent results were observed in the colony formation assay, which showed an obviously increasing number of FHC clones, while a decreasing number of clones were observed in SW480 and LoVo cells (Figure 1B). In addition, The transwell migration assay revealed that calcitriol reduced the number of migrating SW480 and LoVo cells by 38 and 63%, respectively, compared with the vehicle (Figure 1C).

Next, the oncogenicity of CRC cells was determined *in vivo* by subcutaneously injecting SW480 cells into the left back of nude mice. Tumors treated with calcitriol were 54% smaller and 65% lighter than those treated with the vehicle (*p* < 0.05, Figures 1D–F), meaning that calcitriol treatment significantly enhanced tumor growth regression. IHC staining for Ki67 further demonstrated that proliferating cells in tumors treated with calcitriol were much fewer (Figure 1G). Collectively, the above results indicated that 1,25(OH)_2_D_3_ suppressed the aggressive phenotype of CRC both *in vitro* and *in vivo*, suggesting that 1,25(OH)_2_D_3_ might play a suppressive role in CRC progression.

### Predicting the Key Targets of Vitamin D_3_ Against CRC Based on a Network Pharmacological Analysis

**FIGURE 2:**
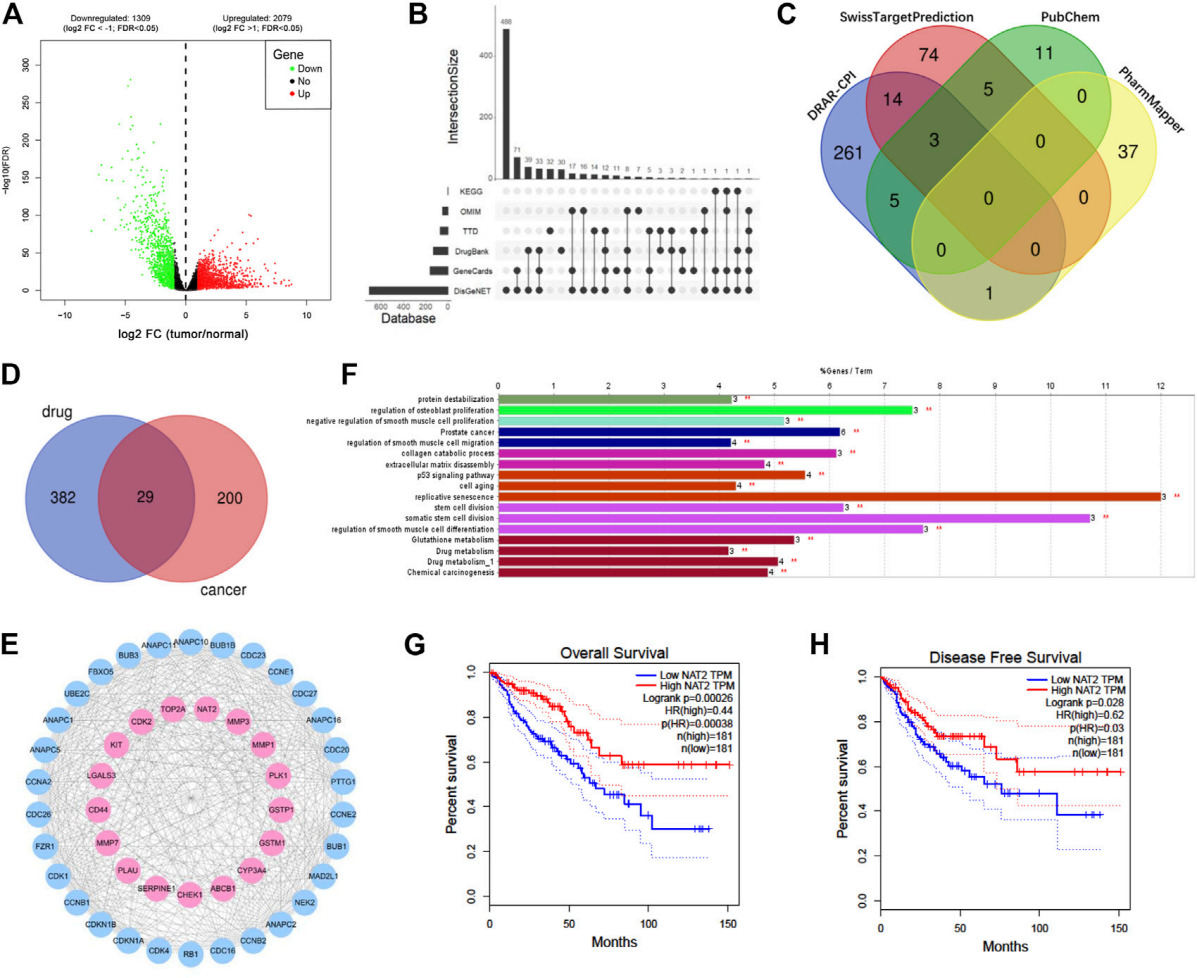
NAT2 is a potential target of vitamin D_3_ against CRC based on a network pharmacological analysis. **(A)** Volcano map of 3388 DEGs (including 2,079 upregulated genes and 1,309 downregulated genes) from TCGA. Red: upregulation; green: downregulation; log2 FC: log2 fold change. **(B)** UpSet plot showing predicted cancer target genes among DEGs by six databases. **(C)** Venn plot showing 411 predicted vitamin D_3_ targets from 4 databases. **(D)** Venn plot showing 29 overlapping targets between vitamin D_3_– and CRC-related genes. **(E)** PPI network of drug cancer targets. Pink: overlapping targets; blue: amplified targets. **(F)** Functionally enriched GO terms and KEGG pathway analysis of the overlapping targets. The bars represent the percentage of associated genes per term. **(G**,**H)** The correlation between NAT2 expression and overall survival **(G)** or disease-free survival **(H)** in CRC patients from GEPIA.

To predict the potential genes that play pivotal roles in CRC, DEGs were screened out from TCGA-CRC expression profile and intersected with targets from other six online databases. The expression of each gene was analyzed and compared between 44 normal samples and 568 CRC samples from TCGA using R software and the edgeR package, and DEGs were extracted with the standard of fold change >2 and FDR < 0.05. Consequently, a total of 3388 DEGs were obtained from TCGA database, in which 2,079 genes were upregulated and 1,309 genes were downregulated (Figure 2A). CRC potential target genes were predicted from six online databases, and genes that existed in at least two databases as long as belonging to DEGs from TCGA database were chosen for further analysis (Figure 2B). Moreover, vitamin D_3_–related target genes were predicted via four online databases, and 411 targets were obtained in total (Figure 2C). KEGG pathway enrichment analysis of CRC and vitamin D_3_–related targets was carried out to primarily understand the functions of these potential targets (Supplementary Figure S1A). Then, intersection analysis between 229 CRC potential target genes and 411 vitamin D_3_–related target genes was carried out, and 29 target genes were overlapped (Figure 2D).

To uncover potential pharmacological mechanisms of vitamin D_3_ against CRC, a network of overlapping genes and amplified targets was built using the STRING database and Cytoscape software (Figure 2E). Furthermore, GO and KEGG functional enrichment analyses of the network were carried out by ClueGO in Cytoscape software (Figure 2F, Supplementary Figure S1B). The correlation between the expression of each overlapping gene and the prognosis of CRC was analyzed by GEPIA. The overall and disease-free survival rates of patients with high NAT2 expression were both higher than those of patients with low NAT2 expression (Figures 2G,H). In addition, the overall survival rate of patients with high LGALS4 expression was higher than that of patients with low LGALS4 expression, and the disease-free survival rate of patients with high CASP7 expression was higher than that of patients with low CASP7 expression (Supplementary Figure S1C). No significant correlation was found between the expression of other overlapping genes and CRC survival. The aforementioned results suggested that NAT2 might play a key role in the interaction of vitamin D_3_ and CRC.

### NAT2 Expression Prominently Declines in CRC Specimens and Correlates With the Clinicopathologic Characteristics of CRC Patients

**FIGURE 3:**
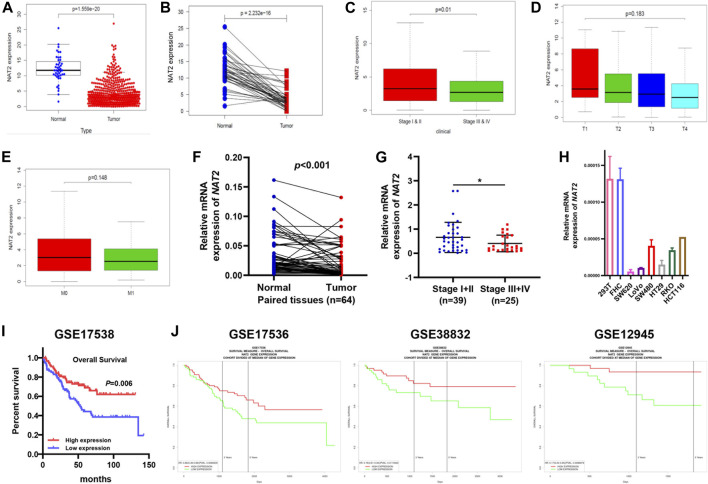
Differentiated expression of NAT2 specimens and its correlation with clinicopathologic characteristics in CRC patients. **(A)** Differentiated expression of NAT2 in colorectal normal and tumor specimens from TCGA. **(B)** Paired differentiation analysis for the expression of NAT2 in 44 matched normal and tumor samples derived from the TCGA-CRC expression profile. **(C**–**E)** The correlation between NAT2 expression and pathological staging characteristics. **(F**,**G)** Differentiated expression of NAT2 in 64 CRC samples and paired adjacent tissues obtained from our hospital (Nanfang Hospital) **(F)** and the correlation between NAT2 expression and AJCC stage **(G)**. **(H)** NAT2 mRNA expression in normal and CRC cell lines. **(I**,**J)** Survival analysis of CRC patients with different NAT2 expression levels from GSE17538, GSE17536, GSE38832, and GSE12945. [Student’s *t* test for **(A**–**C**,**E**–**G)**; One-way ANOVA for **(D)**; log-rank test for **(I)**. *, *p* < 0.05].

To explore the expression profiling of NAT2 in CRC, 612 CRC samples from TCGA were used for further analysis. The Wilcoxon rank-sum test revealed that the expression of NAT2 in tumor samples was significantly lower than that in normal samples (Figure 3A). Pairing analysis between the normal and tumor samples derived from the same patients showed a consistent result (Figure 3B). In addition, the American Joint Committee on Cancer (AJCC) tumor-node-metastasis (TNM) classification is a globally recognized standard for cancer staging. According to the clinical stage, 612 samples were divided into two groups (AJCC stages I and II and stages III and IV) and NAT2 expression was calculated, respectively, and compared between groups. Obviously, the expression of NAT2 was significantly decreased with the progression of the clinical stage in CRC (Figure 3C). Similar analyses were carried out on the level of T stage and M stage. The mean value of NAT2 expression showed a downward trend with the progression of T stage and M stage in CRC, even though the results were lacking statistical significance (Figures 3D,E). A decrease in NAT2 expression was also observed in most CRC datasets by Oncomine dataset analysis (Supplementary Figure S1D). In addition, the expression profiling of NAT2 was confirmed by 64 pairs of fresh CRC specimens (Figures 3F,G). The chi-square test indicated that NAT2 expression was negatively related to tumor differentiation and AJCC stage in CRC (*p* = 0.03 and *p* = 0.02, respectively; Table 1), which was confirmed by GSE17538 expression profiling (Supplementary Table S2). Moreover, NAT2 mRNA expression in CRC cell lines was obviously lower than that in normal cell lines (Figure 3H).

**TABLE 1:** Correlation between the expression of NAT2 and clinicopathologic characteristics.

Clinicopathologic characteristic	NAT2 expression		*p*-value
	High (*n* = 32)	Low (*n* = 32)		
**Age (years)**			
<65	22	18	0.30
≥65	10	14	
**Gender**			
Male	18	21	0.44
Female	14	11	
**Tumor**			
Well-moderate	26	18	**0.03**
Poor	6	14	
**AJCC stage**			
I-II	24	15	**0.02**
III-IV	8	17	

The bold values refer to p < 0.05.

**FIGURE 4:**
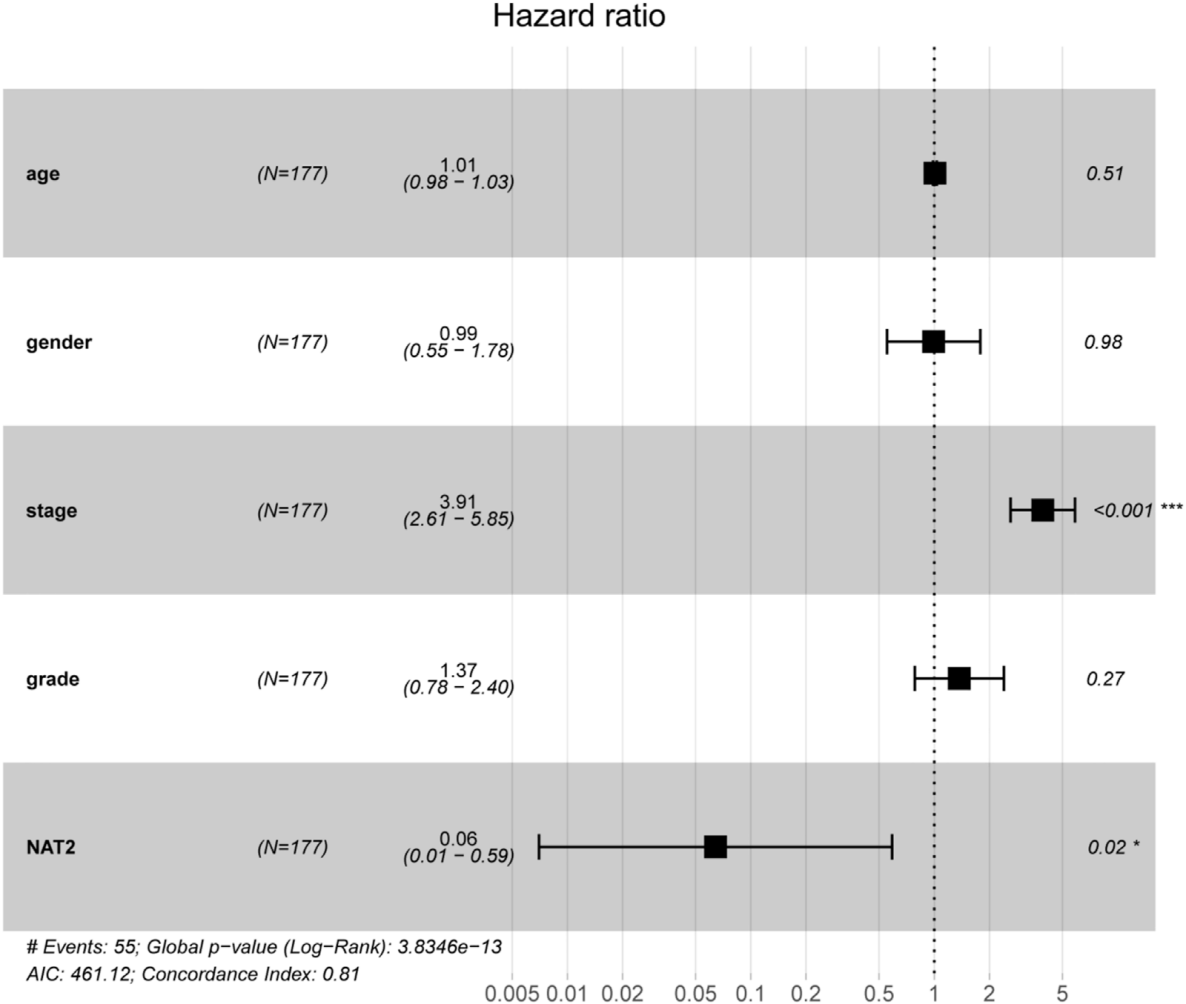
Prognostic value of NAT2 in CRC patients based on a forest plot analysis. Clinical features (age, gender, stage, and grade) and NAT2 expression were analyzed to assess the hazard ratio for CRC patients. *, *p* < 0.05; ***, *p* < 0.001.

To verify the correlation between NAT2 expression and the prognosis of CRC patients, 4 cohorts (GSE17538, GSE17536, GSE38832, and GSE12945) were used for survival analysis. The survival curves showed that CRC patients with high NAT2 expression had a better prognosis than those with low NAT2 expression (Figures 3I,J), which were consistent with the results from the GEPIA-CRC expression profile. Furthermore, a forest plot was constructed to display the hazard ratio of NAT2 and clinical features (age, gender, stage, and grade), which suggested that NAT2 could be an independent clinical risk factor in CRC (hazard ratio for death, 0.06; 95% CI, 0.01 to 0.59; *p* = 0.02; Figure 4).

### NAT2 Inhibits the Malignant Phenotype of CRC Cells and Negatively Regulates the JAK1/STAT3 Signaling Pathway

**FIGURE 5:**
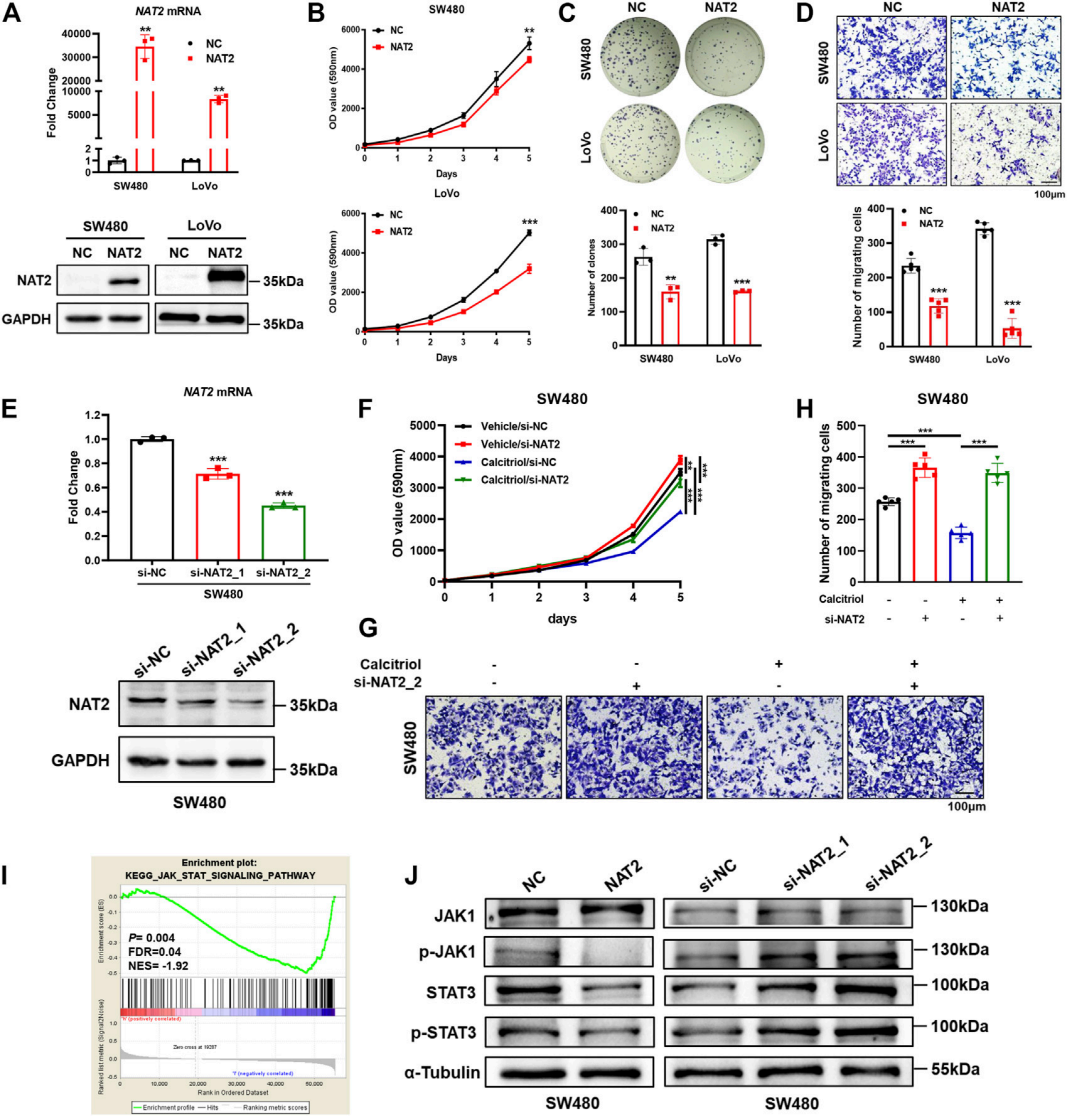
NAT2 suppresses the proliferation and migration of CRC cells and negatively regulates the JAK1/STAT3 signaling pathway. **(A)** The expression of NAT2 was verified by RT-qPCR and Western blot assays in SW480 and LoVo cells transfected with the negative control or NAT2-overexpressing plasmid. **(B**–**D)** Cell proliferation assay **(B)**, colony formation assay **(C)**, and transwell migration assay **(D)** in the negative control or NAT2-overexpressing CRC cells. Scale bar: 100 μm. **(E)** RT-qPCR and Western blot assays in SW480 cells transfected with the negative control or NAT2-targeting siRNA. **(F**–**H)** Cell proliferation assay **(F)** and transwell migration assay **(G**,**H)** in negative control or si-NAT2 SW480 cells treated with the vehicle or 100 nM calcitriol. Scale bar: 100 μm. **(I)** GSEA indicated that the expression of NAT2 was negatively correlated with the JAK/STAT signaling pathway gene signatures (data source: TCGA). **(J)** Western blot assays for key molecules of the JAK/STAT signaling pathway in SW480 cells transfected with the NAT2-overexpressing plasmid or NAT2-targeting siRNA. Three independent experiments were performed for **(A**–**H**,**J)**. The data are presented as mean ± SD (Student’s *t* test. **, *p* < 0.01; ***, *p* < 0.001).

To investigate the biological function of NAT2 in CRC cells, SW480 and LoVo cells were transfected with the negative control or NAT2-overexpressing plasmid. A significant increase in NAT2 mRNA and protein expression in SW480 and LoVo cells was observed by RT-qPCR and Western blot assays (Figure 5A, Supplementary Figure S2A). Remarkably, overexpression of NAT2 decreased the growth rate and number of clones in SW480 and LoVo cells compared with the negative control group (Figures 5B,C). Additionally, transwell migration assays revealed a weaker migration ability in NAT2-overexpressing CRC cells (Figure 5D).

Furthermore, negative control or NAT2-targeting siRNA was transfected into SW480 cells, and decreased expression of NAT2 was verified by RT-qPCR and Western blot assays (Figure 5E, Supplementary Figure S2B). Then si-NAT2_2 SW480 cells were used to perform cell proliferation assays and transwell migration assays with or without calcitriol treatment. There was an obvious increase in the growth rate and more migrated cells in si-NAT2_2 SW480 cells than in si-NC SW480 cells (Figures 5F–H). As mentioned before (Figures 1A,C), calcitriol impaired the proliferation and invasiveness of CRC cells, while transfecting NAT2-targeting siRNA could regain the aggressive phenotype of CRC cells. In summary, these results revealed that calcitriol suppresses the malignancy of CRC cells via NAT2. To explore the regulation of NAT2 in the cancer signaling pathway, TCGA data were analyzed by GSEA (Supplementary Figure S2C). The JAK/STAT signaling pathway was significantly downregulated by NAT2 (normalized enrichment score (NES) = −1.92, FDR = 0.04; Figure 5I), which was confirmed by Western blot assays in SW480 cells (Figure 5J, Supplementary Figures S2D,E).

### 1,25(OH)_2_D_3_ Activates VDR and Upregulates NAT2 *in vitro* and *in vivo*

**FIGURE 6:**
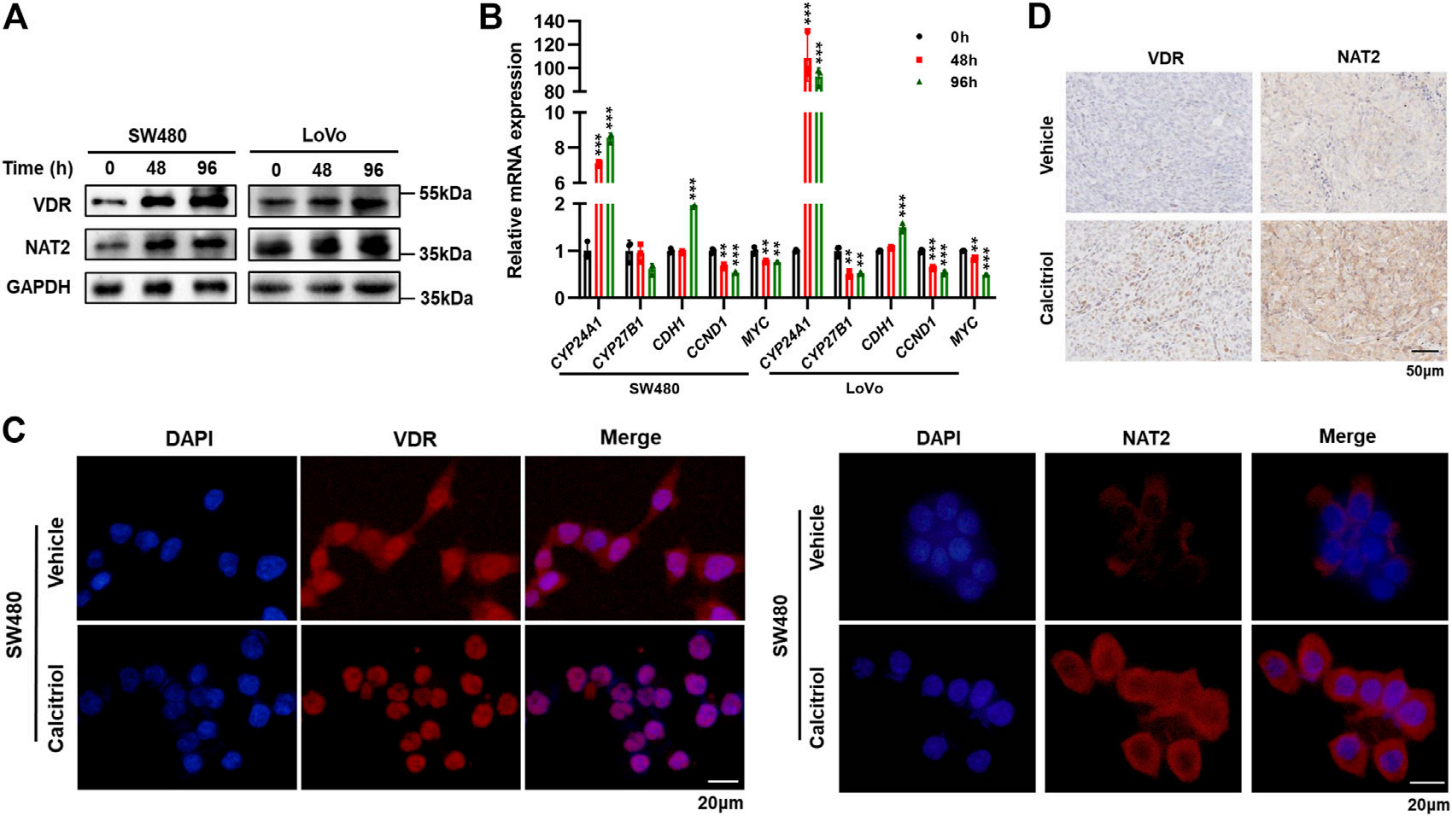
1,25(OH)_2_D_3_ induces VDR nuclear translocation and promotes NAT2 expression *in vitro* and *in vivo*. **(A)** Western blot assays in SW480 and LoVo cells treated with 100 nM calcitriol for 0, 48, or 96 h **(B)** RT-qPCR analysis of CYP24A1, CYP27B1, CDH1, CCND1, and MYC mRNA in SW480 and LoVo cells treated with calcitriol or vehicle for the indicated times. **(C)** Immunofluorescence staining assays for DAPI (blue) and VDR (red, left) or NAT2 (red, right) in SW480 cells treated with the vehicle or 100 nM calcitriol for 48 h. Scale bar: 20 μm. **(D)** Representative images of IHC staining for VDR and NAT2 in subcutaneous tumors. Scale bar: 50 μm. Three independent experiments were performed for **(A**–**C)**. The data are presented as mean ± SD (Student’s *t* test. **, *p* < 0.01; ***, *p* < 0.001).

To investigate whether 1,25(OH)_2_D_3_ regulates NAT2, Western blot assays were performed in SW480 and LoVo cells treated with 100 nM calcitriol for 0, 48, or 96 h. The results showed a time-dependent increase in the expression of VDR and NAT2 (Figure 6A, Supplementary Figure S3A). Two key enzymes (CYP24A1 and CYP27B1) involved in vitamin D metabolism were validated by RT-qPCR to confirm VDR activation in SW480 and LoVo cells in response to calcitriol (Feldman et al., 2014; Porter et al., 2019). Moreover, a key factor involved in epithelial to mesenchymal transition (CDH1) was induced, and cell cycle-related factors (CCND1 and MYC) were repressed in cells treated with calcitriol (Figure 6B). Immunofluorescence staining assays revealed that calcitriol stimulation induced VDR nuclear translocation and increased NAT2 expression (Figure 6C). In addition, IHC staining of subcutaneous tumors showed that both VDR and NAT2 were upregulated in subcutaneous tumors from the calcitriol treatment group (Figure 6D). According to the aforementioned analyses, 1,25(OH)_2_D_3_ could induce VDR nuclear translocation and upregulate NAT2 *in vitro* and *in vivo*.

### VDR Upregulates NAT2 by Binding to NAT2 Promoter

**FIGURE 7:**
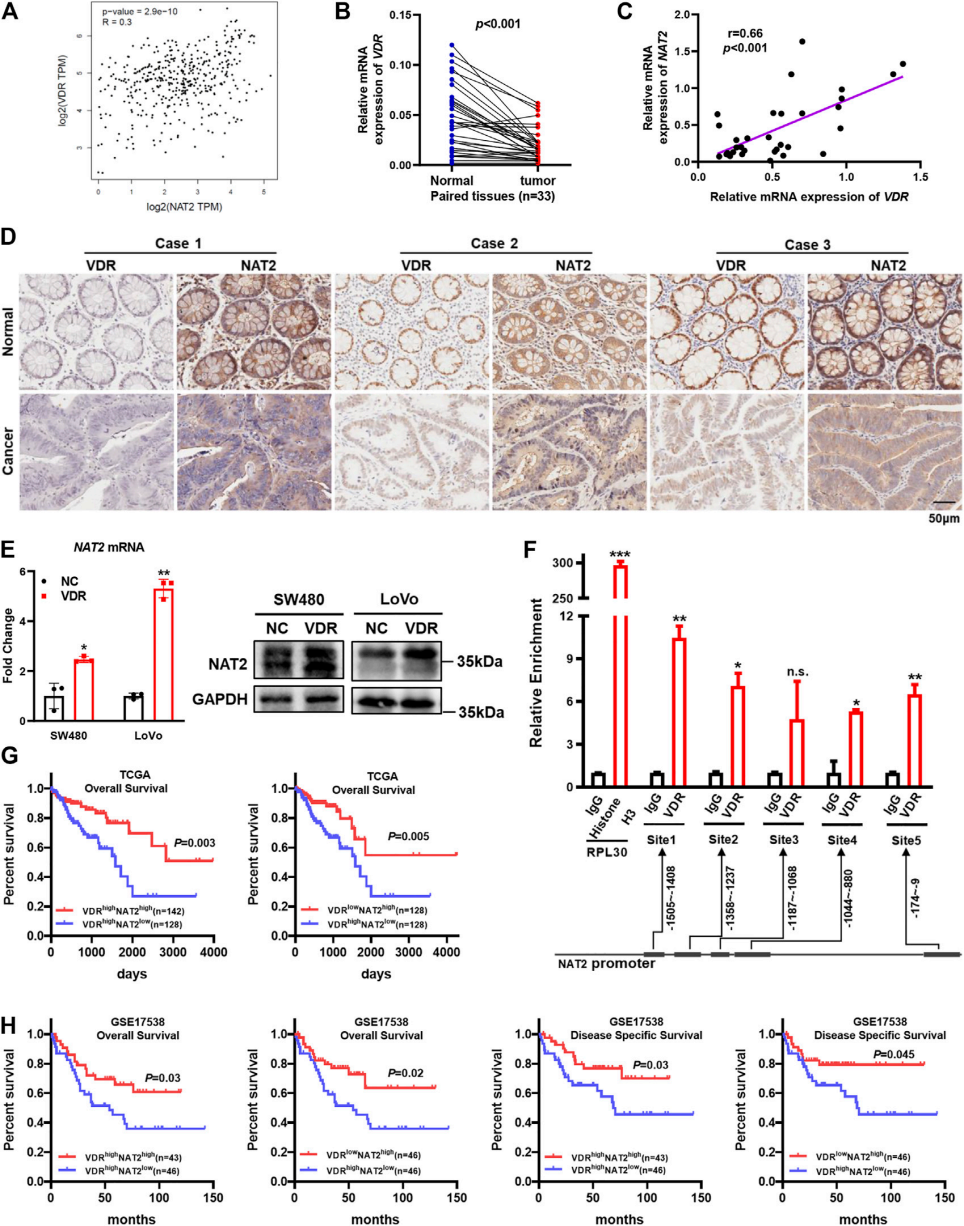
VDR promotes NAT2 expression by binding to the NAT2 promoter. **(A)** Correlation between NAT2 and VDR mRNA expression in normal colorectal tissues and CRC tissues from GEPIA. **(B**,**C)** VDR mRNA expression was detected in 33 consecutive CRC specimens and paired adjacent normal specimens **(B)** and was positively correlated with NAT2 **(C)**. **(D)** IHC staining in serial sections of paired CRC tissues was performed to analyze the correlation between VDR and NAT2 protein expression. Three typical cases among 19 patients with CRC are displayed. Scale bar: 50 μm. **(E)** RT-qPCR and Western blot assays of NAT2 in SW480 and LoVo cells transfected with the negative control or VDR overexpression plasmid. **(F)** Chromatin immunoprecipitation assay was used to confirm that VDR binds to the promoter region of NAT2 in SW480 cells. The immunoprecipitated DNA was quantified by RT-qPCR. **(G**,**H)** Kaplan–Meier survival curves depicting the survival of patients with CRC stratified by VDR and NAT2 mRNA expression levels [data sources: TCGA **(G)** and GSE17538 **(H)**]. Three independent experiments were performed for **(E)**. The data are presented as mean ± SD [Spearman’s correlation analysis for **(A**,**C)**; Student’s *t* test for **(B**,**E**,**F)**; Log-rank test for **(G**,**H)**. *, *p* < 0.05; **, *p* < 0.01; ***, *p* < 0.001; n.s., non-significant].

Human clinical data from the GEPIA showed a modest positive correlation between NAT2 and VDR mRNA expression (Figure 7A). Compared with paired adjacent normal colorectal tissues, the VDR mRNA expression markedly decreased in 33 consecutive CRC tissues among the 64 aforementioned fresh specimens from Nanfang Hospital (Figure 7B), and correlation analysis verified a close relation between VDR and NAT2 (*r* = 0.66, *p* < 0.001; Figure 7C). In addition, IHC staining displayed lower expression of VDR and NAT2 in tumor tissues than matched normal tissues and simultaneous alteration of the two proteins in 19 cases, among which three typical cases were displayed (Figure 7D).

To investigate whether VDR could regulate NAT2, the VDR overexpression plasmid was transfected into SW480 and LoVo cells, and RT-qPCR, and Western blot assays verified that VDR was overexpressed successfully (Supplementary Figures S3B,C). Moreover, both the mRNA and protein expression of NAT2 notably increased in VDR-overexpressing CRC cells compared with the negative control (Figure 7E, Supplementary Figure S3D). In addition, VDR-overexpressing CRC cells exhibited a slower growth rate and weaker migration capacity (Supplementary Figures S3E–G). VDR regulates downstream targets by binding to the promoter regions of target genes. Thus, we performed the chromatin immunoprecipitation assay in VDR-overexpressing SW480 cells to analyze the regulatory effects of VDR on the transcription of NAT2. The immunoprecipitated DNA was quantified by RT-qPCR, and the result indicated that the NAT2 promoter region exhibited significant enrichment in four VDR-binding sites after immunoprecipitation with an anti-VDR antibody (Figure 7F). Interestingly, data from TCGA and GSE17538 demonstrated that patients with both high VDR expression and low NAT2 expression had the worst prognosis (Figures 7G,H). This indicated that the NAT2 expression level may be more vital to predict prognosis when the transcriptional regulation of VDR is disturbed in CRC.

## Discussion

To date, increasing epidemiological evidence has indicated the positive role of vitamin D in cancer prevention, especially in colorectal cancer. Populations with lower circulating vitamin D concentration are at higher risk of CRC (Garland et al., 1985; Jenab et al., 2010), and vitamin D deficiency predicts shortened overall survival in CRC patients (Ng et al., 2008; Zgaga et al., 2014). Remarkably, a recent randomized controlled trial revealed that vitamin D_3_ can reduce the incidence rate of advanced cancer, especially in individuals with normal BMI (Chandler et al., 2020). Another prospective clinical trial aimed to determine whether high-dose vitamin D_3_ could improve outcomes in patients undergoing standard chemotherapy for metastatic CRC. The results showed a difference in median progression-free survival, although it was not significant, and the risk of progression decreased by high-dose vitamin D_3_ versus standard-dose vitamin D_3_ (Ng et al., 2019). The aforementioned clinical trials verify that vitamin D_3_ inhibits colorectal carcinogenesis and increases sensitivity to chemotherapy. Furthermore, numerous studies have successively come to similar conclusions by *in vitro* and *in vivo* experiments (Padi et al., 2013; Razak et al., 2019; Shang et al., 2020; Aslam et al., 2021). A notable study in pancreatic cancer showed that vitamin D_3_ increased gemcitabine concentration in tumor tissues; thus, tumors were smaller and animals survived longer than when gemcitabine alone was given (Sherman Mara et al., 2014). Nevertheless, the underlying mechanisms demonstrating the protective effects of vitamin D_3_ in CRC are not completely understood. In the present study, we certified that 1,25(OH)_2_D_3_ suppresses proliferation, clonogenicity, and invasion activity of CRC cells and restricts the growth of cell-derived xenografts. Interestingly, supplementation with 1,25(OH)_2_D_3_ promoted the proliferation of the human normal colorectal cell line (FHC).

Network pharmacological analysis is a potent method to integrate “Big Data” and comprehensively elaborate the underlying molecular mechanisms (Ma'ayan et al., 2014). According to individual genetic background, the method can increase drug therapeutic efficacy and reduce invalid treatment. We, by tumor and drug databases, identified 29 intersecting potential targets correlated with vitamin D_3_ against CRC. Subsequent pathway enrichment analysis for intersecting genes elaborated the underlying molecular mechanisms, including stem cell division, replicative senescence, p53 signaling pathway, and chemical carcinogenesis. Among the 29 intersecting genes, NAT2 is the only one that correlated robustly with both overall survival and disease-free survival. Moreover, database analysis and our experimental results showed that NAT2 was substantially downregulated in CRC and negatively correlated with the AJCC stage, which indicates that NAT2 may act as a tumor suppressor gene in CRC.

NAT2, expressed in colon and liver tissues principally, catalyzes the acetylation of arylamines and is involved in the metabolism of carcinogens (Hickman et al., 1998). On account of the N-acetyltransferase enzyme variant, NAT2 is classified as slow and rapid acetylator (Ilett et al., 1994). Association studies have revealed that NAT2 polymorphisms are related to susceptibility to cancer (Hengstler et al., 1998; Agúndez, 2008; Lin et al., 2009). Previous epidemiological studies declared the NAT2 rapid acetylator phenotype as a hazardous factor for cancer, especially CRC, in subjects exposed to meat and tobacco heterocyclic amines (Hein et al., 1993; Chen et al., 1998; Chan et al., 2005; Nöthlings et al., 2009; Andersen et al., 2013; Voutsinas et al., 2013), but others proposed that it was a protective factor (Hubbard et al., 1997; Heinimann et al., 1999). Meanwhile, there are some studies that demonstrated that the NAT2 genotype did not modify the positive correlation between red meat intake or heterocyclic amines and CRC risk (Ananthakrishnan et al., 2015; Budhathoki et al., 2015). In addition, the NAT2 genotype may affect the age-associated risk of hereditary non-polyposis CRC and play an important role in tumorigenesis among individuals with mismatch repair defects (Heinimann et al., 1999; Frazier et al., 2001). A noteworthy study concluded that microRNA-217–targeting NAT2 restrains proliferation and promotes apoptosis and autophagy in rat models of CCL4-induced liver injury (Yang et al., 2019). Another study proposed that microRNA-6477-5p increased anoikis sensitivity by targeting NAT1 (an important paralog of NAT2) in breast cancer (Malagobadan et al., 2020). Moreover, the NAT1 mRNA level is associated with the overall survival of breast cancer patients and may be induced to identify non-responders to chemotherapy (Minchin and Butcher, 2018).

The aforementioned studies indicate that NAT2 may contribute to the progression of CRC and other diseases. Thus far, it remains unclear that how NAT2 affects carcinogenesis in CRC. Here, we preliminarily elaborate that NAT2 prevents malignant biological behavior of CRC cells and inhibits the JAK1/STAT3 signaling pathway in CRC cells. The JAK/STAT signaling pathway is well-known for its imperative role in cancer and among promising therapeutic targets. As a versatile pathway, its canonical effects are promoting tumor cell proliferation, survival, invasion, and immunosuppression (Yu et al., 2014). However, this is just a crude description of the role of NAT2 in CRC carcinogenesis, which is expected to achieve further clarification in detail.

High VDR expression in CRC tissues was reported to be associated with a better clinical outcome (Ferrer-Mayorga et al., 2017; Hu et al., 2020). VDR is a transcription factor that has complex and pleiotropic effects on the regulation of biological processes. For example, 1,25(OH)_2_D_3_ suppresses proliferation by inducing microRNA-627, which subsequently targets JMJD1A and suppresses the expression of cancer-promoting genes such as GDF15 (Padi et al., 2013). Additionally, a recent report states that VDR restricts stemness and chemotherapy resistance by regulating SOX2 (Hu et al., 2020), a factor that maintains tumor-initiating cells, and is associated with poor prognosis. For stromal cells, 1,25(OH)_2_D_3_ can inhibit the protumoral activation of fibroblasts and impair the migration capacity of CRC cells (Ferrer-Mayorga et al., 2017). Our results show that the expression of NAT2 is strongly related to that of VDR in CRC clinical samples, and 1,25(OH)_2_D_3_ induces the expression of NAT2 by transcriptional regulation of VDR. Thus, siRNA-targeting endogenous NAT2 counteracts the antineoplastic effects of 1,25(OH)_2_D_3_ in CRC cells. Notably, data from TCGA and GEO-CRC expression profiles reveal that the NAT2 expression level may be more critical to predict prognosis when the transcriptional regulation of VDR is disturbed. Judging from the results, we infer that nonresponse to vitamin D_3_ may be due to abnormal NAT2 expression in CRC patients. Genotype-based NAT2 activity might lead to interindividual variability in sensitivity to vitamin D_3_ in CRC, but further study to elaborate the detailed molecular mechanism is warranted.

**FIGURE 8:**
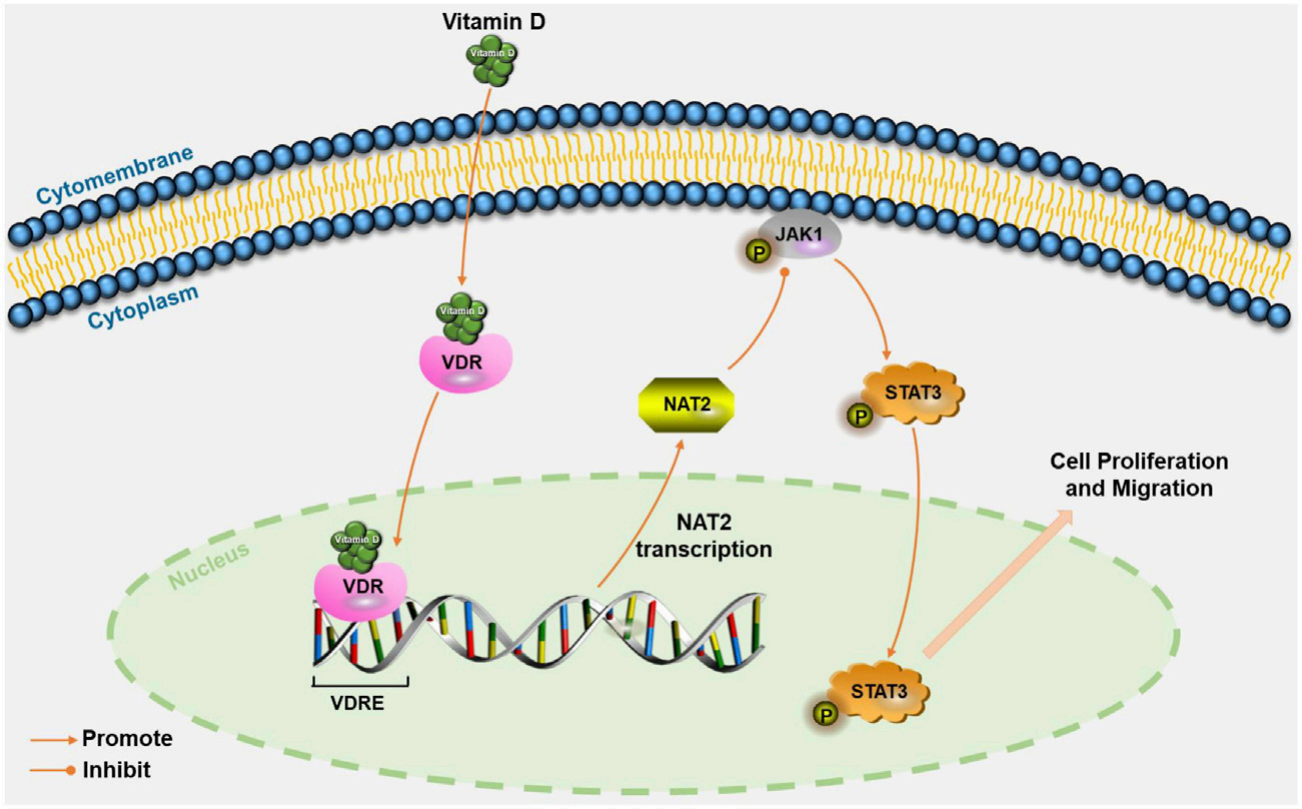
Schematic illustration of VDR signaling via NAT2 in CRC cells. Vitamin D induces NAT2 expression via transcriptional regulation of VDR, and NAT2 acts as a tumor suppressor factor via the JAK1/STAT3 signaling pathway. VDRE: vitamin D response elements.

In conclusion, we identified NAT2 as a key target of vitamin D_3_ against CRC by network pharmacology analysis and molecular biology experiments. We confirmed that 1,25(OH)_2_D_3_ upregulates NAT2 via transcriptional regulation of VDR and restrains the progression of CRC by *in vitro* and *in vivo* experiments. NAT2 was found to be downregulated in CRC specimens, and the lower expression of NAT2 was correlated with a higher metastasis risk and lower survival rate of CRC patients. Moreover, we proposed that NAT2 suppressed the malignant cellular behavior of CRC cells, perhaps through the JAK1/STAT3 signaling pathway (Figure 8). Collectively, this work verifies that 1,25(OH)_2_D_3_ represses colorectal carcinogenesis and introduces VDR signaling via NAT2 as a potential diagnostic and therapeutic target for CRC.

## Data Availability

The datasets presented in this study can be found in online repositories. The names of the repository/repositories and accession number(s) can be found in the article/Supplementary Material.

## References

[B1] Agúndez J. A. (2008). Polymorphisms of Human N-Acetyltransferases and Cancer Risk. Curr. Drug Metab. 9 (6), 520–531. 10.2174/138920008784892083 18680472

[B2] Ananthakrishnan A. N. Du M. Berndt S. I. Brenner H. Caan B. J. Casey G. (2015). Red Meat Intake, NAT2, and Risk of Colorectal Cancer: a Pooled Analysis of 11 Studies. Cancer Epidemiol. Biomarkers Prev. 24 (1), 198–205. 10.1158/1055-9965.epi-14-0897 25342387 PMC4294960

[B3] Andersen V. Holst R. Vogel U. (2013). Systematic Review: Diet-Gene Interactions and the Risk of Colorectal Cancer. Aliment. Pharmacol. Ther. 37 (4), 383–391. 10.1111/apt.12180 23216531 PMC3565452

[B4] Aslam A. Ahmad J. Baghdadi M. A. Idris S. Almaimani R. Alsaegh A. (2021). Chemopreventive Effects of Vitamin D(3) and its Analogue, Paricalcitol, in Combination with 5-fluorouracil against Colorectal Cancer: The Role of Calcium Signalling Molecules. Biochim. Biophys. Acta Mol. Basis Dis. 1867 (3), 166040. 10.1016/j.bbadis.2020.166040 33338596

[B5] Barry E. L. Peacock J. L. Rees J. R. Bostick R. M. Robertson D. J. Bresalier R. S. (2017). Vitamin D Receptor Genotype, Vitamin D3 Supplementation, and Risk of Colorectal Adenomas: A Randomized Clinical Trial. JAMA Oncol. 3 (5), 628–635. 10.1001/jamaoncol.2016.5917 27978548 PMC5580351

[B6] Bhasin N. Alleyne D. Gray O. A. Kupfer S. S. (2018). Vitamin D Regulation of the Uridine Phosphorylase 1 Gene and Uridine-Induced DNA Damage in Colon in African Americans and European Americans. Gastroenterology 155 (4), 1192–1204. 10.1053/j.gastro.2018.06.049 29964038 PMC6866230

[B7] Brenner H. Kloor M. Pox C. P. (2014). Colorectal Cancer. Lancet 383 (9927), 1490–1502. 10.1016/s0140-6736(13)61649-9 24225001

[B8] Budhathoki S. Iwasaki M. Yamaji T. Sasazuki S. Takachi R. Sakamoto H. (2015). Dietary Heterocyclic Amine Intake, NAT2 Genetic Polymorphism, and Colorectal Adenoma Risk: the Colorectal Adenoma Study in Tokyo. Cancer Epidemiol. Biomarkers Prev. 24 (3), 613–620. 10.1158/1055-9965.epi-14-1051 25604583

[B9] Chan A. T. Tranah G. J. Giovannucci E. L. Willett W. C. Hunter D. J. Fuchs C. S. (2005). Prospective Study of N-Acetyltransferase-2 Genotypes, Meat Intake, Smoking and Risk of Colorectal Cancer. Int. J. Cancer 115 (4), 648–652. 10.1002/ijc.20890 15700302

[B10] Chandler P. D. Chen W. Y. Ajala O. N. Hazra A. Cook N. Bubes V. (2020). Effect of Vitamin D3 Supplements on Development of Advanced Cancer: A Secondary Analysis of the VITAL Randomized Clinical Trial. JAMA Netw. Open 3 (11), e2025850. 10.1001/jamanetworkopen.2020.25850 33206192 PMC7675103

[B11] Chen J. Stampfer M. J. Hough H. L. Garcia-Closas M. Willett W. C. Hennekens C. H. (1998). A Prospective Study of N-Acetyltransferase Genotype, Red Meat Intake, and Risk of Colorectal Cancer. Cancer Res. 58 (15), 3307–3311. 9699660

[B12] Feldman D. Krishnan A. V. Swami S. Giovannucci E. Feldman B. J. (2014). The Role of Vitamin D in Reducing Cancer Risk and Progression. Nat. Rev. Cancer 14 (5), 342–357. 10.1038/nrc3691 24705652

[B13] Ferrer-Mayorga G. Gómez-López G. Barbáchano A. Fernández-Barral A. Peña C. Pisano D. G. (2017). Vitamin D Receptor Expression and Associated Gene Signature in Tumour Stromal Fibroblasts Predict Clinical Outcome in Colorectal Cancer. Gut 66 (8), 1449–1462. 10.1136/gutjnl-2015-310977 27053631 PMC5530491

[B14] Ferrer-Mayorga G. Larriba M. J. Crespo P. Muñoz A. (2019). Mechanisms of Action of Vitamin D in colon Cancer. J. Steroid Biochem. Mol. Biol. 185, 1–6. 10.1016/j.jsbmb.2018.07.002 29981368

[B15] Frazier M. L. O'Donnell F. T. Kong S. Gu X. Campos I. Luthra R. (2001). Age-associated Risk of Cancer Among Individuals with N-Acetyltransferase 2 (NAT2) Mutations and Mutations in DNA Mismatch Repair Genes. Cancer Res. 61 (4), 1269–1271. 11245417

[B16] Garland C. F. Garland F. C. (1980). Do sunlight and Vitamin D Reduce the Likelihood of colon Cancer? Int. J. Epidemiol. 9 (3), 227–231. 10.1093/ije/9.3.227 7440046

[B17] Garland C. Shekelle R. B. Barrett-Connor E. Criqui M. H. Rossof A. H. Paul O. (1985). Dietary Vitamin D and Calcium and Risk of Colorectal Cancer: a 19-year Prospective Study in Men. Lancet 1 (8424), 307–309. 10.1016/s0140-6736(85)91082-7 2857364

[B18] Hein D. W. Doll M. A. Gray K. Rustan T. D. Ferguson R. J. (1993). Metabolic Activation of N-Hydroxy-2-Aminofluorene and N-Hydroxy-2-Acetylaminofluorene by Monomorphic N-Acetyltransferase (NAT1) and Polymorphic N-Acetyltransferase (NAT2) in colon Cytosols of Syrian Hamsters Congenic at the NAT2 Locus. Cancer Res. 53 (3), 509–514. 8425184

[B19] Heinimann K. Scott R. J. Chappuis P. Weber W. Müller H. Dobbie Z. (1999). N-acetyltransferase 2 Influences Cancer Prevalence in hMLH1/hMSH2 Mutation Carriers. Cancer Res. 59 (13), 3038–3040. 10397239

[B20] Hengstler J. G. Arand M. Herrero M. E. Oesch F. (1998). Polymorphisms of N-Acetyltransferases, Glutathione S-Transferases, Microsomal Epoxide Hydrolase and Sulfotransferases: Influence on Cancer Susceptibility. Recent Results Cancer Res. 154, 47–85. 10.1007/978-3-642-46870-4_4 10026993

[B21] Hickman D. Pope J. Patil S. D. Fakis G. Smelt V. Stanley L. A. (1998). Expression of Arylamine N-Acetyltransferase in Human Intestine. Gut 42 (3), 402–409. 10.1136/gut.42.3.402 9577349 PMC1727045

[B22] Hopkins A. L. (2008). Network Pharmacology: the Next Paradigm in Drug Discovery. Nat. Chem. Biol. 4 (11), 682–690. 10.1038/nchembio.118 18936753

[B23] Hu P. S. Li T. Lin J. F. Qiu M. Z. Wang D. S. Liu Z. X. (2020). VDR-SOX2 Signaling Promotes Colorectal Cancer Stemness and Malignancy in an Acidic Microenvironment. Signal. Transduct Target. Ther. 5 (1), 183. 10.1038/s41392-020-00230-7 32900990 PMC7479104

[B24] Hubbard A. L. Harrison D. J. Moyes C. Wyllie A. H. Cunningham C. Mannion E. (1997). N-acetyltransferase 2 Genotype in Colorectal Cancer and Selective Gene Retention in Cancers with Chromosome 8p Deletions. Gut 41 (2), 229–234. 10.1136/gut.41.2.229 9301503 PMC1891458

[B25] Ilett K. F. Ingram D. M. Carpenter D. S. Teitel C. H. Lang N. P. Kadlubar F. F. (1994). Expression of Monomorphic and Polymorphic N-Acetyltransferases in Human colon. Biochem. Pharmacol. 47 (5), 914–917. 10.1016/0006-2952(94)90493-6 8135868

[B26] Jenab M. Bueno-de-Mesquita H. B. Ferrari P. van Duijnhoven F. J. Norat T. Pischon T. (2010). Association between Pre-diagnostic Circulating Vitamin D Concentration and Risk of Colorectal Cancer in European Populations:a Nested Case-Control Study. Bmj 340, b5500. 10.1136/bmj.b5500 20093284 PMC2809840

[B27] Ji M. T. Nie J. Nie X. F. Hu W. T. Pei H. L. Wan J. M. (2020). 1α,25(OH)(2)D(3) Radiosensitizes Cancer Cells by Activating the NADPH/ROS Pathway. Front. Pharmacol. 11, 945. 10.3389/fphar.2020.00945 32848720 PMC7426479

[B28] Lai Q. Li Q. He C. Fang Y. Lin S. Cai J. (2020). CTCF Promotes Colorectal Cancer Cell Proliferation and Chemotherapy Resistance to 5-FU via the P53-Hedgehog axis. Aging (Albany NY) 12 (16), 16270–16293. 10.18632/aging.103648 32688344 PMC7485712

[B29] Lamprecht S. A. Lipkin M. (2003). Chemoprevention of colon Cancer by Calcium, Vitamin D and Folate: Molecular Mechanisms. Nat. Rev. Cancer 3 (8), 601–614. 10.1038/nrc1144 12894248

[B30] Laversanne M. Soerjomataram I. Jemal A. Bray F. (2020). Colorectal Cancer Statistics, 2020. CA Cancer J. Clin. 64, 104–117. 10.3322/caac.21601

[B31] Lichtenstein P. Holm N. V. Verkasalo P. K. Iliadou A. Kaprio J. Koskenvuo M. (2000). Environmental and Heritable Factors in the Causation of Cancer-Aanalyses of Cohorts of Twins from Sweden, Denmark, and Finland. N. Engl. J. Med. 343 (2), 78–85. 10.1056/nejm200007133430201 10891514

[B32] Lin J. Kamat A. Gu J. Chen M. Dinney C. P. Forman M. R. (2009). Dietary Intake of Vegetables and Fruits and the Modification Effects of GSTM1 and NAT2 Genotypes on Bladder Cancer Risk. Cancer Epidemiol. Biomarkers Prev. 18 (7), 2090–2097. 10.1158/1055-9965.epi-08-1174 19549811

[B33] Luo X. Fong E. L. S. Zhu C. Lin Q. X. X. Xiong M. Li A. (2021). Hydrogel-based Colorectal Cancer Organoid Co-culture Models. Acta Biomater. 132, 461–472. 10.1016/j.actbio.2020.12.037 33388439

[B34] Ma'ayan A. Rouillard A. D. Clark N. R. Wang Z. Duan Q. Kou Y. (2014). Lean Big Data Integration in Systems Biology and Systems Pharmacology. Trends Pharmacol. Sci. 35 (9), 450–460. 10.1016/j.tips.2014.07.001 25109570 PMC4153537

[B35] Malagobadan S. Ho C. S. Nagoor N. H. (2020). MicroRNA-6744-5p Promotes Anoikis in Breast Cancer and Directly Targets NAT1 Enzyme. Cancer Biol. Med. 17 (1), 101–111. 10.20892/j.issn.2095-3941.2019.0010 32296579 PMC7142838

[B36] McCullough M. L. Zoltick E. S. Weinstein S. J. Fedirko V. Wang M. Cook N. R. (2019). Circulating Vitamin D and Colorectal Cancer Risk: An International Pooling Project of 17 Cohorts. J. Natl. Cancer Inst. 111 (2), 158–169. 10.1093/jnci/djy087 29912394 PMC6376911

[B37] Minchin R. F. Butcher N. J. (2018). Trimodal Distribution of Arylamine N-Acetyltransferase 1 mRNA in Breast Cancer Tumors: Association with Overall Survival and Drug Resistance. BMC Genomics 19 (1), 513. 10.1186/s12864-018-4894-4 29969986 PMC6029418

[B38] Ng K. Meyerhardt J. A. Wu K. Feskanich D. Hollis B. W. Giovannucci E. L. (2008). Circulating 25-hydroxyvitamin D Levels and Survival in Patients with Colorectal Cancer. J. Clin. Oncol. 26 (18), 2984–2991. 10.1200/jco.2007.15.1027 18565885

[B39] Ng K. Nimeiri H. S. McCleary N. J. Abrams T. A. Yurgelun M. B. Cleary J. M. (2019). Effect of High-Dose vs Standard-Dose Vitamin D3 Supplementation on Progression-free Survival Among Patients with Advanced or Metastatic Colorectal Cancer: The SUNSHINE Randomized Clinical Trial. Jama 321 (14), 1370–1379. 10.1001/jama.2019.2402 30964527 PMC6459117

[B40] Ng K. Sargent D. J. Goldberg R. M. Meyerhardt J. A. Green E. M. Pitot H. C. (2011). Vitamin D Status in Patients with Stage IV Colorectal Cancer: Findings from Intergroup Trial N9741. J. Clin. Oncol. 29 (12), 1599–1606. 10.1200/jco.2010.31.7255 21422438 PMC3082978

[B41] Nöthlings U. Yamamoto J. F. Wilkens L. R. Murphy S. P. Park S. Y. Henderson B. E. (2009). Meat and Heterocyclic Amine Intake, Smoking, NAT1 and NAT2 Polymorphisms, and Colorectal Cancer Risk in the Multiethnic Cohort Study. Cancer Epidemiol. Biomarkers Prev. 18 (7), 2098–2106. 10.1158/1055-9965.epi-08-1218 19549810 PMC2771770

[B42] Padi S. K. Zhang Q. Rustum Y. M. Morrison C. Guo B. (2013). MicroRNA-627 Mediates the Epigenetic Mechanisms of Vitamin D to Suppress Proliferation of Human Colorectal Cancer Cells and Growth of Xenograft Tumors in Mice. Gastroenterology 145 (2), 437–446. 10.1053/j.gastro.2013.04.012 23619147 PMC3722307

[B43] Porter R. L. Magnus N. K. C. Thapar V. Morris R. Szabolcs A. Neyaz A. (2019). Epithelial to Mesenchymal Plasticity and Differential Response to Therapies in Pancreatic Ductal Adenocarcinoma. Proc. Natl. Acad. Sci. U S A. 116 (52), 26835–26845. 10.1073/pnas.1914915116 PMC6936349 31843922

[B44] Razak S. Afsar T. Almajwal A. Alam I. Jahan S. (2019). Growth Inhibition and Apoptosis in Colorectal Cancer Cells Induced by Vitamin D-Nanoemulsion (NVD): Involvement of Wnt/beta-Catenin and Other Signal Transduction Pathways. Cell Biosci 9, 15. 10.1186/s13578-019-0277-z 30733856 PMC6359839

[B45] Shang J. Zhu Z. Chen Y. Song J. Huang Y. Song K. (2020). Small-molecule Activating SIRT6 Elicits Therapeutic Effects and Synergistically Promotes Anti-tumor Activity of Vitamin D3 in Colorectal Cancer. Theranostics 10 (13), 5845–5864. 10.7150/thno.44043 32483423 PMC7255010

[B46] Sherman Mara H. Yu Ruth T. Engle Dannielle D. Ding N. Atkins Annette R. Tiriac H. (2014). Vitamin D Receptor-Mediated Stromal Reprogramming Suppresses Pancreatitis and Enhances Pancreatic Cancer Therapy. Cell 159 (1), 80–93. 10.1016/j.cell.2014.08.007 25259922 PMC4177038

[B47] Sung H. Ferlay J. Siegel R. L. Laversanne M. Soerjomataram I. Jemal A. (2021). Global Cancer Statistics 2020: GLOBOCAN Estimates of Incidence and Mortality Worldwide for 36 Cancers in 185 Countries. CA Cancer J. Clin. 71 (3), 209–249. 10.3322/caac.21660 33538338

[B48] Voutsinas J. Wilkens L. Franke A. Vogt T. Yokochi L. Decker R. (2013). Heterocyclic Amine Intake, Smoking, Cytochrome P450 1A2 and N-Acetylation Phenotypes, and Risk of Colorectal Adenoma in a Multiethnic Population. Gut 62 (3), 416–422. 10.1136/gutjnl-2011-300665 22628494 PMC4491437

[B49] Wang K. X. Gao Y. Lu C. Li Y. Zhou B. Y. Qin X. M. (2020). Uncovering the Complexity Mechanism of Different Formulas Treatment for Rheumatoid Arthritis Based on a Novel Network Pharmacology Model. Front. Pharmacol. 11, 1035. 10.3389/fphar.2020.01035 32754034 PMC7365894

[B50] Wu X. Hu W. Lu L. Zhao Y. Zhou Y. Xiao Z. (2019). Repurposing Vitamin D for Treatment of Human Malignancies via Targeting Tumor Microenvironment. Acta Pharm. Sin B. 9 (2), 203–219. 10.1016/j.apsb.2018.09.002 30972274 PMC6437556

[B51] Yang C. L. Zheng X. L. Ye K. Sun Y. N. Lu Y. F. Ge H. (2019). Effects of microRNA-217 on Proliferation, Apoptosis, and Autophagy of Hepatocytes in Rat Models of CCL4-Induced Liver Injury by Targeting NAT2. J. Cel Physiol. 234 (4), 3410–3424. 10.1002/jcp.26748 30417525

[B52] Yu H. Lee H. Herrmann A. Buettner R. Jove R. (2014). Revisiting STAT3 Signalling in Cancer: New and Unexpected Biological Functions. Nat. Rev. Cancer 14 (11), 736–746. 10.1038/nrc3818 25342631

[B53] Zgaga L. Theodoratou E. Farrington S. M. Din F. V. Ooi L. Y. Glodzik D. (2014). Plasma Vitamin D Concentration Influences Survival Outcome after a Diagnosis of Colorectal Cancer. J. Clin. Oncol. 32 (23), 2430–2439. 10.1200/jco.2013.54.5947 25002714

