# Supplementary figures and images for: VDR Signaling via the Enzyme NAT2 Inhibits Colorectal Cancer Progression
Source: Front Pharmacol. 2021 Nov 16;12:727704. doi: 10.3389/fphar.2021.727704 (PMC8635240; doi:10.3389/fphar.2021.727704)

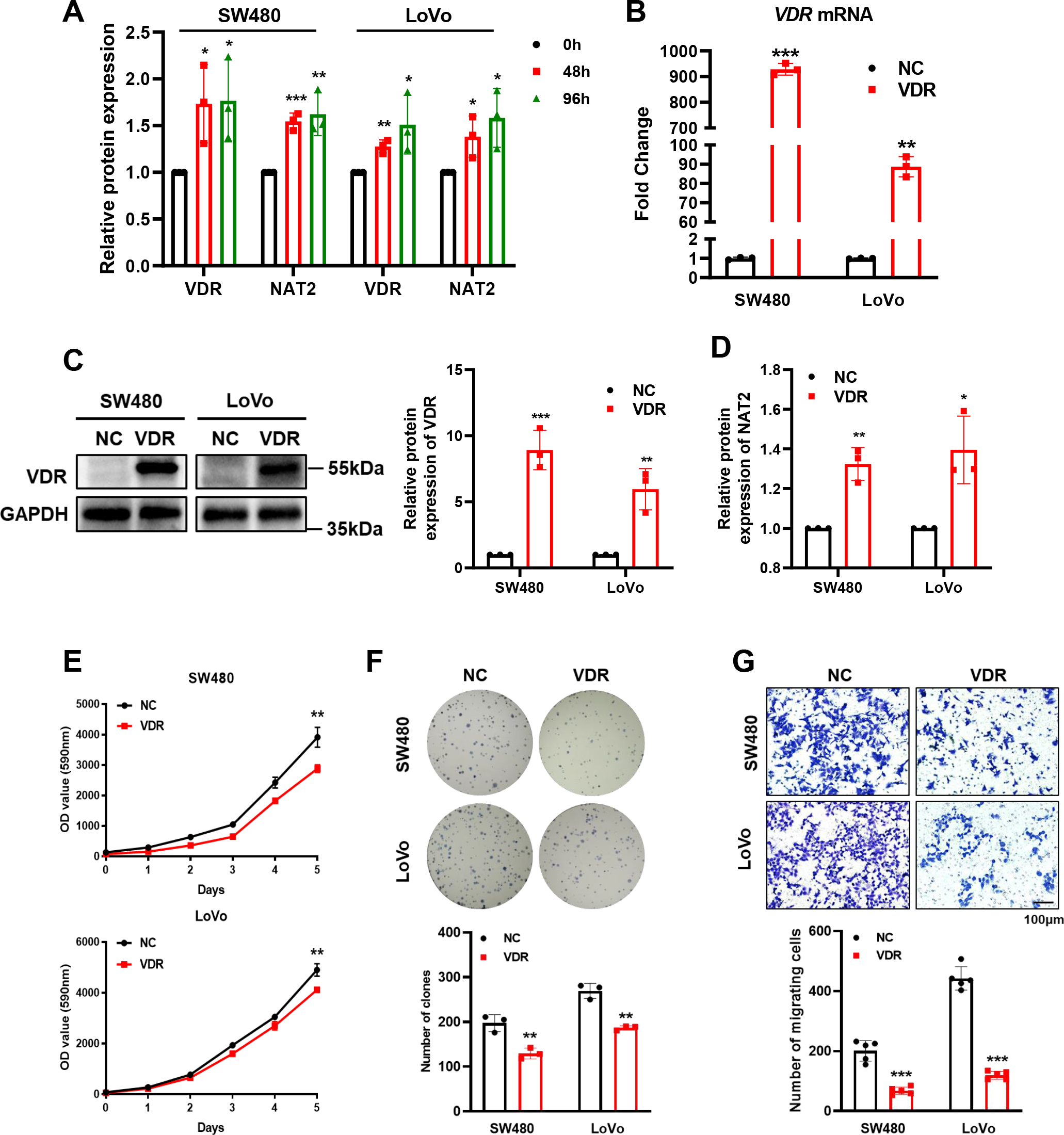

Supplement: Supplementary file 1 [file Image3.TIF]

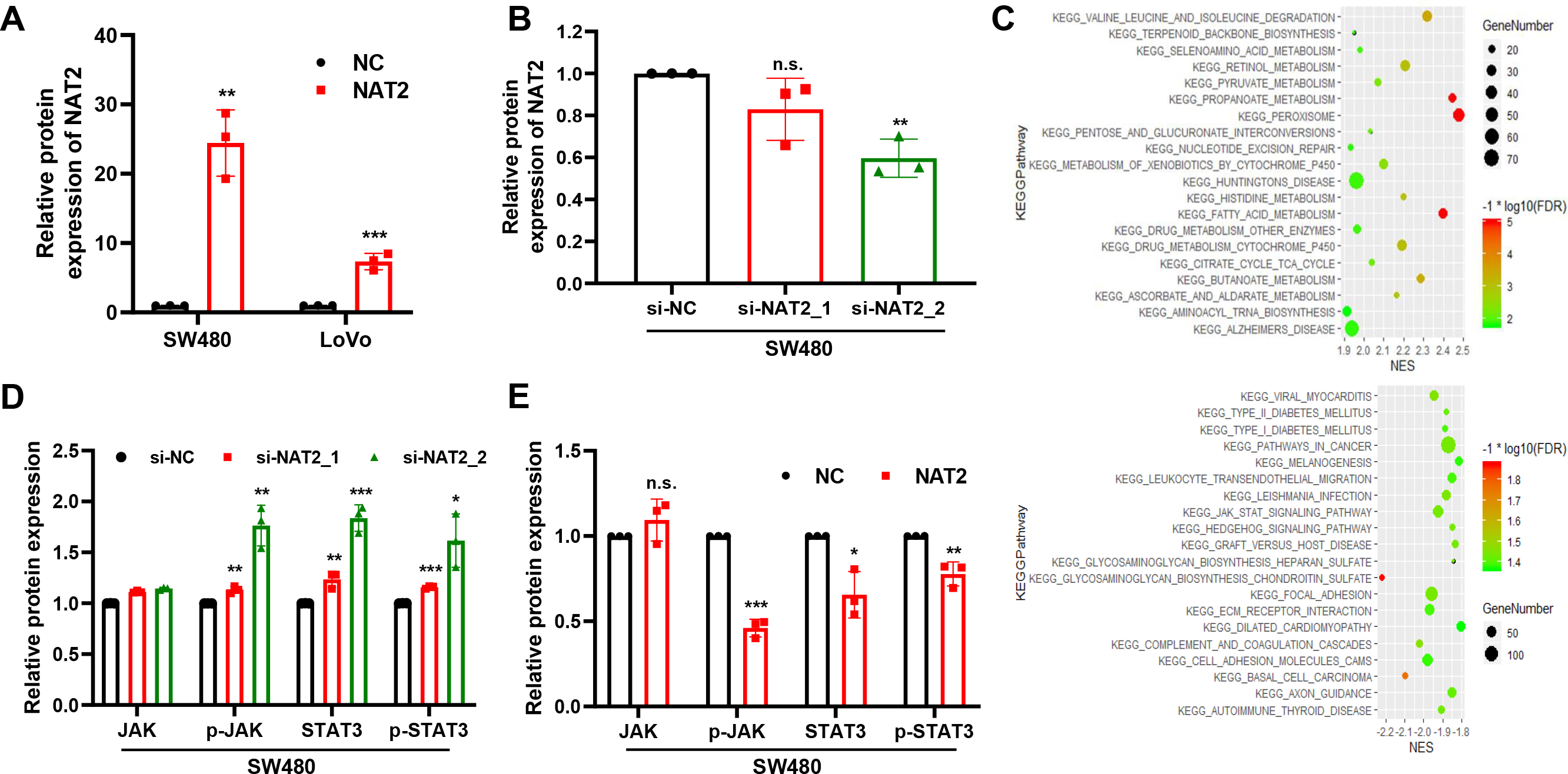

Supplement: Supplementary file 3 [file Image2.TIF]

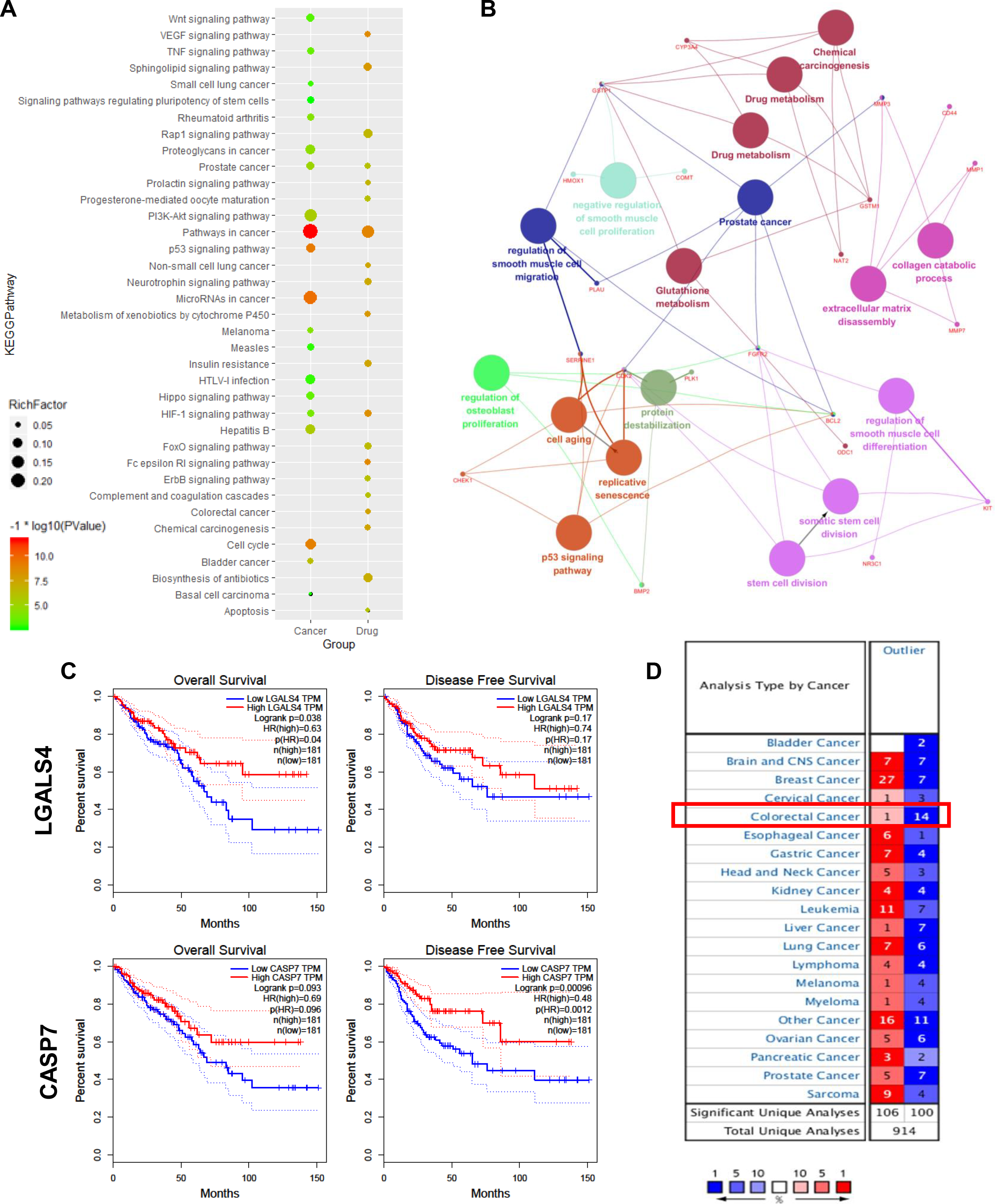

Supplement: Supplementary file 4 [file Image1.TIF]
