# Supplementary material for: VDR Signaling via the Enzyme NAT2 Inhibits Colorectal Cancer Progression
Source: Front Pharmacol. 2021 Nov 16;12:727704. doi: 10.3389/fphar.2021.727704 (PMC8635240; doi:10.3389/fphar.2021.727704)
Supplement: Supplementary file 2 [file DataSheet3.docx]

Supplementary Material

# Supplementary Tables

**Supplementary Table S1.** Sequence information used in this study

| siRNA | Sense | antisense |
| --- | --- | --- |
| si-NAT2­_1 | 5’-GGGCUGUUCCCUUUGAGAATT-3’ | 5’-UUCUCAAAGGGAACAGCCCTT-3’ |
| si-NAT2­_2 | 5’-GCAGCCUCUAGAAUUAAUUTT-3’ | 5’-AAUUAAUUCUAGAGGCUGCTT-3’ |
| negative control | 5’-UUCUCCGAACGUGUCACGUTT-3’ | 5’-ACGUGACACGUUCGGAGAATT-3’ |
| Gene | **Forward** | **Reverse** |
| *GAPDH* | 5'-CTGGGCTACACTGAGCACC-3' | 5'-AAGTGGTCGTTGAGGGCAATG-3' |
| *NAT2* | 5'-AGTTGGGCTTAGAGGCTATTTT-3' | 5'-ATTGTGGTCTGAAAACCGATTG-3' |
| *VDR* | 5’-GTGGACATCGGCATGATGAAG-3’ | 5’-GGTCGTAGGTCTTATGGTGGG-3’ |
| NAT2-ChIP (-1502~-1408) | 5’-CAGGAGTAGGAGGCAATCAAA-3’ | 5’-TTTGTCTTGTGGCCTGGTTTT-3’ |
| NAT2-ChIP (-1358~-1237) | 5’-CACCAGTGCGGGAGTATAACA-3’ | 5’-ACTGCCTCCTGGTGTTGTCTA-3’ |
| NAT2-ChIP (-1187~-1068) | 5’-AAGAAAGCCTTCCCACAGAGT-3’ | 5’-GCCTTTAGCACCTACTGTTCC-3’ |
| NAT2-ChIP (-1044~-880) | 5’-TGGAACAGTAGGTGCTAAAGG-3’ | 5’-CTAGCTTCCATACCCTCTTTT-3’ |
| NAT2-ChIP (-174~-9) | 5’-GCATGGGTATCATCAGATAATATAA-3’ | 5’-TTCCAAAGTCTGCAAGGGAAG-3’ |

**Supplementary Table S2.** Correlation between the expression of NAT2 and clinicopathological characteristics (GES17538)

| **Clinicopathological parameters** | ***NAT2* expression** | | **Total** | **P-value** |
| --- | --- | --- | --- | --- |
|  | **High (n = 89)** | **Low (n = 88)** |  |  |
| **Age** |  |  |  |  |
| ＜65 years | 40 (51.3) | 38 (48.7) | 78 | 0.81 |
| ≥65 years | 49 (49.5) | 50 (50.5) | 99 |  |
| **Gender** |  |  |  |  |
| Male | 49 (51.0) | 47 (49.0) | 96 | 0.83 |
| Female | 40 (49.4) | 41 (50.6) | 81 |  |
| **Tumor differentiation** |  |  |  |  |
| Well-moderate | 82 (55.0) | 67 (45.0) | 149 | 0.004 |
| Poor | 7 (25.0) | 21 (75.0) | 28 |  |
| **Pathological stage** |  |  |  |  |
| I-II | 48 (59.3) | 33 (40.7) | 81 | 0.03 |
| III-IV | 41 (42.7) | 55 (57.3) | 96 |  |

# Supplementary Figures

**Supplementary Figure S1.** Identifying the potential key targets of vitamin D_3_ against CRC via bioinformatics analysis. **(A)** Bubble chart showing the result of KEGG pathway enrichment analysis of 229 CRC potential targets and 411 vitamin D_3_ related targets. **(B)** Functionally enriched GO terms and KEGG pathways analysis of overlapping targets. **(C)** The overall and disease-free survival of *LGALS4* and *CASP7* expression in CRC patients from GEPIA. **(D)** *NAT2* expression was investigated by Oncomine database.

**Supplementary Figure S2.** NAT2 negatively regulates the JAK/STAT3 signaling pathway. **(A, B)** The relative intensity values of NAT2 as described in Figure 5A and 5E were measured with ImageJ software. **(C)** GSEA for top 20 enriched pathways upregulated (upper) and downregulated (lower) by NAT2 (data source: TCGA). **(D, E)** The relative intensity values of JAK1, p-JAK1, STAT3 and p-STAT3 as described in Figure 5J. (Student’s t test. *, *p* < 0.05; **, *p* < 0.01; ***, *p* < 0.001; n.s., nonsignificant)

**Supplementary Figure S3. (A)** The relative intensity values of VDR and NAT2 as described in Figure 6A. **(B, C)** The expression of VDR was verified by RT-qPCR and western blot assays in SW480 and LoVo cells transfected with negative control or VDR overexpression plasmid. **(D)** The relative intensity values of NAT2 as described in Figure 7E. **(E-G)** Cell proliferation assay (E), colony formation assay (F) and Transwell migration assay (G) in negative control or VDR-overexpressing CRC cells. Scale bar: 100 μm. (Student’s t test. *, *p* < 0.05; **, *p* < 0.01; ***, *p* < 0.001).
